# Rab22a-NeoF1 fusion protein promotes osteosarcoma lung metastasis through its secretion into exosomes

**DOI:** 10.1038/s41392-020-00414-1

**Published:** 2021-02-11

**Authors:** Li Zhong, Dan Liao, Jingjing Li, Wenqiang Liu, Jingxuan Wang, Cuiling Zeng, Xin Wang, Zhiliang Cao, Ruhua Zhang, Miao Li, Kuntai Jiang, Yi-Xin Zeng, Jianhua Sui, Tiebang Kang

**Affiliations:** 1 State Key Laboratory of Oncology in South China, Collaborative Innovation Center for Cancer Medicine, Sun Yat-sen University Cancer Center, Guangzhou, China; 2 Department of Oncology, The Fifth Affiliated Hospital of Sun Yat-Sen University, Zhuhai, Guangdong China; 3 National Institute of Biological Sciences, Beijing, China; 4 Sun Yat-sen University School of Medicine, Shenzhen, China; 5 Zhongshan School of Medicine, Sun Yat-sen University, Guangzhou, China

**Keywords:** Metastasis, Bone cancer

## Abstract

It remains unknown for decades how some of the therapeutic fusion proteins positive in a small percentage of cancer cells account for patient outcome. Here, we report that osteosarcoma Rab22a-NeoF1 fusion protein, together with its binding partner PYK2, is sorted into exosomes by HSP90 via its KFERQ-like motif (RVLFLN^142^). The exosomal Rab22a-NeoF1 fusion protein facilitates the pulmonary pre-metastatic niche formation by recruiting bone marrow-derived macrophages. The exosomal PYK2 activates RhoA in its negative recipient osteosarcoma cells and induces signal transducer and activator of transcription 3 activation in its recipient macrophages to increase M2 phenotype. Consequently, lung metastases of its recipient osteosarcoma cells are promoted by this exosomal Rab22a-NeoF1 fusion protein, and this event can be targeted by disrupting its interaction with PYK2 using a designed internalizing RGD peptide.

## Introduction

Osteosarcoma is one of the most common malignant bone cancers with a high peak of incidence in adolescents.^1^ The majority of osteosarcoma patients suffer from non-metastatic disease, and up to 70% survive with local surgery combined with neoadjuvant multidrug chemotherapy.^2,3^ The 5-year overall survival still remains only 20% over the past 30 years since 15–30% patients had already presented lung metastasis at diagnosis.^4^ Redundancy in growth signals and high heterogeneity lead to no effective molecular therapeutic targets in osteosarcoma.^5^ Our group has just identified a new fusion gene termed *RAB22A*-NeoF1, which is derived from chromosomal translocations that juxtapose amino acids 1–38 of the trafficking-related Rab22a with inverted intron sequences of *DOK5*. The Rab22a-NeoF1 fusion protein drives lung metastasis of osteosarcoma by activating RhoA through constitutively binding to a negatively charged region of SmgGDS607, and may be a potential target for patients with osteosarcoma metastases.^6^

Extracellular vesicles (EVs) are considered as a critical mechanism for cell-to-cell communication.^7^ Exosomes are 30–150 nm diameter vesicles derived from intraluminal vesicles and are released during fusion of multivesicular endosome or multivesicular bodies with plasma membrane^8^ through a well-defined intracellular trafficking route.^9–13^ Tumor-derived exosomes carrying numerous bioactive cargos, including lipids, proteins, and regulatory RNAs, can be taken up by neighboring cells or enter the circulation and arrive at distant sites to influence behavior of recipient cells.^14,15^ Tumor exosomes can transport regulatory RNAs that promote chemoresistance in breast cancer and renal cancer,^16,17^ oncoproteins that enhance the motility of cancer cells^18–21^ or facilitate pre-metastatic niche formation,^22–24^ and membrane proteins that mediate immunosuppression.^25^ Tumor-derived exosomes can be detected in biological fluid and serve as a potential diagnostic marker and may predict response to therapy.^26^ Therefore, we surmised that any fusion protein that is positive in a small percentage of cancer cells may present in exosomes, which may be taken up by their surrounding cells, such as macrophages and cancer cells negative for the fusion gene, to affect the entire cancer progression, as it remains unknown for decades how fusion protein accounts for patient outcome.

In this study, we found that the osteosarcoma Rab22a-NeoF1 fusion protein is secreted into exosomes by binding to HSP90 via its KFERQ-like motif, which is taken up by macrophages and cancer cells negative for this fusion gene, and that the exosomal Rab22a-NeoF1 fusion protein can promote its negative recipient cancer cells to metastasize to lungs in mice through the activation of RhoA by its binding partner PYK2 from donor cells.

## Results

### Rab22a-NeoF1 fusion protein is secreted into media and enhances lung metastasis

**Fig. 1:**
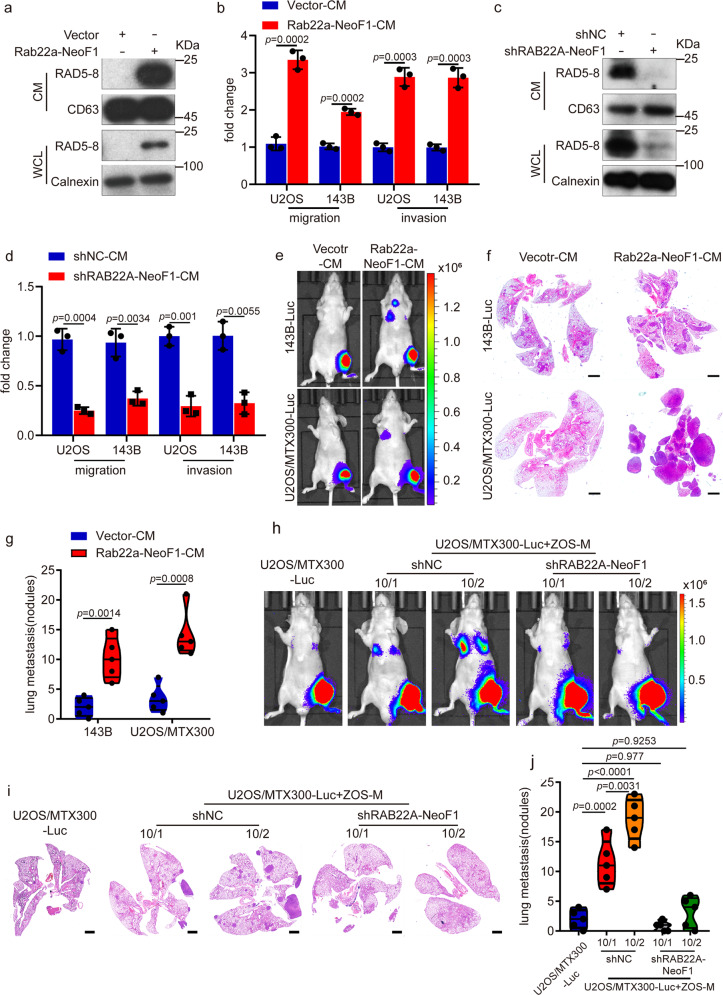
The secreted Rab22a-NeoF1 fusion protein promotes metastasis in osteosarcoma. **a**, **c** The conditioned media (CM) and cell lysates (WCL) derived from the indicated stable 143B cells (**a**) and ZOS-M cells (**c**) were prepared and analyzed by Western blotting. Data in **a**, **c** are representative of *n* = 3 biologically independent experiments. **b**, **d** The indicated cells were treated with the conditioned media derived from vector cells (vector-CM), Rab22a-NeoF1 cells (Rab22a-NeoF1-CM), ZOS-M-shNC cells (shNC-CM), or ZOS-M-shRAB22A-NeoF1 cells (shRAB22A-NeoF1-CM) for 24 h and then were subjected to migration and invasion assays. Data are mean ± s.d. of *n* = 3 biologically independent experiments. *P* values are shown. **e**–**g** Representative IVIS imaging (**e**), H&E-stained lung sections (**f**), and quantification of lung metastatic foci (**g**) from mice orthotopically injected with the indicated cells under treatment of either Vector-CM or Rab22a-NeoF1-CM. *n* = 5 biologically independent mice. Data are mean ± s.d. *P* values are shown. Scale bar, 2 mm. **h**–**j** Representative IVIS imaging (**h**), H&E-stained lung sections (**i**), and quantification of lung metastatic foci (**j**) from mice orthotopically co-injected U2OS/MTX300-Luc cells with either ZOS-M-shNC cells or ZOS-M-shRAB22A-NeoF1 cells at the indicated ratios. *n* = 5 biologically independent mice. Data are mean ± s.d. *P* values are shown. Scale bar, 2 mm

Since osteosarcoma has a high heterogeneity, the *RAB22A*-NeoF1 fusion gene we have just reported was positive only in a small proportion of osteosarcoma cells from a clinical sample.^6^ We were curious to ask whether this fusion protein presents in exosomes, which may be taken up by its surrounding cells, such as macrophages and cancer cells negative for this fusion gene, to affect cancer progression, as exosomes play critical roles in the cell-to-cell communication.^7^ Interestingly, Rab22a-NeoF1 fusion protein was detected in the conditioned media (CM) from cells stably expressing Rab22a-NeoF1 (Rab22a-NeoF1-CM), but not from cells stably expressing vector (Vector-CM) (Fig. 1a). Furthermore, cell migration and invasion were significantly enhanced by incubation with Rab22a-NeoF1-CM, but not Vector-CM, in both U2OS and 143B cells that are negative for *RAB22A*-NeoF1 fusion gene (Fig. 1b). Consistently, the promotion of cell migration and invasion was significantly decreased in both U2OS and 143B cells treated with the CM derived from ZOS-M-sh*RAB22A*-NeoF1, ZOS-M cells knockdown endogenous *RAB22A*-NeoF1 using short hairpin RNAs (shRNAs), compared to those derived from ZOS-M-shNC (Fig. 1c, d). Moreover, the injection of Rab22a-NeoF1-CM, but not Vector-CM, promoted lung metastases of mice in the orthotopic osteosarcoma metastasis model in vivo using both 143B-Luc, 143B cells stably expressing luciferase, and U2OS/MTX300-Luc cells, U2OS/MTX300 cells stably expressing luciferase (Fig. 1e–g and Supplementary Fig. 1a).

Next, using the co-culture of 143B-Luc cells with either ZOS-M-shNC or ZOS-M-sh*RAB22A*-NeoF1 at the 1:1 ratio, we also found that cell migration and invasion of 143B cells co-cultured with ZOS-M cells were diminished by knocking down *RAB22A*-NeoF1 in ZOS-M cells (Supplementary Fig. 1b). More importantly, we orthotopically co-injected 143B-Luc cells with either ZOS-M-shNC or ZOS-M-sh*RAB22A*-NeoF1 cells at the 1:1 ratio into mice, and found that lung metastases of 143B-Luc cells were significantly decreased in mice bearing tumors from their mixtures with ZOS-M-sh*RAB22A*-NeoF1 cells compared to those with ZOS-M-shNC cells (Supplementary Fig. 1c–f).

Since Rab22a-NeoF1 only occurs in a subset of cancer cells, we sought to mimic the heterogeneous osteosarcoma tumors in mice by orthotopically injecting U2OS/MTX300-luc cells alone or together with either ZOS-M-shNC or ZOS-M-sh*RAB22A*-NeoF1 at both the 10:1 and 10:2 ratios. The lung metastases of U2OS/MTX300-luc cells were significantly increased in mice bearing tumors from their mixtures with ZOS-M-shNC cells compared to those with ZOS-M-sh*RAB22A*-NeoF1 cells (Fig. 1h–j and Supplementary Fig. 1g). It worth noting that the enhancement in mice with the 10:2 ratio was stronger than those with the 10:1 ratio, indicating that the more lung metastases of negative cancer cells, the more positive cancer cells have in these heterogeneous osteosarcoma tumors (Fig. 1h–j and Supplementary Fig. 1g). Collectively, these results demonstrate that the conditioned medium derived from tumor cells positive for *RAB22A*-NeoF1 promotes its negative osteosarcoma cells to metastasize to lungs.

### Rab22a-NeoF1 fusion protein is secreted into exosomes to promote metastasis

**Fig. 2:**
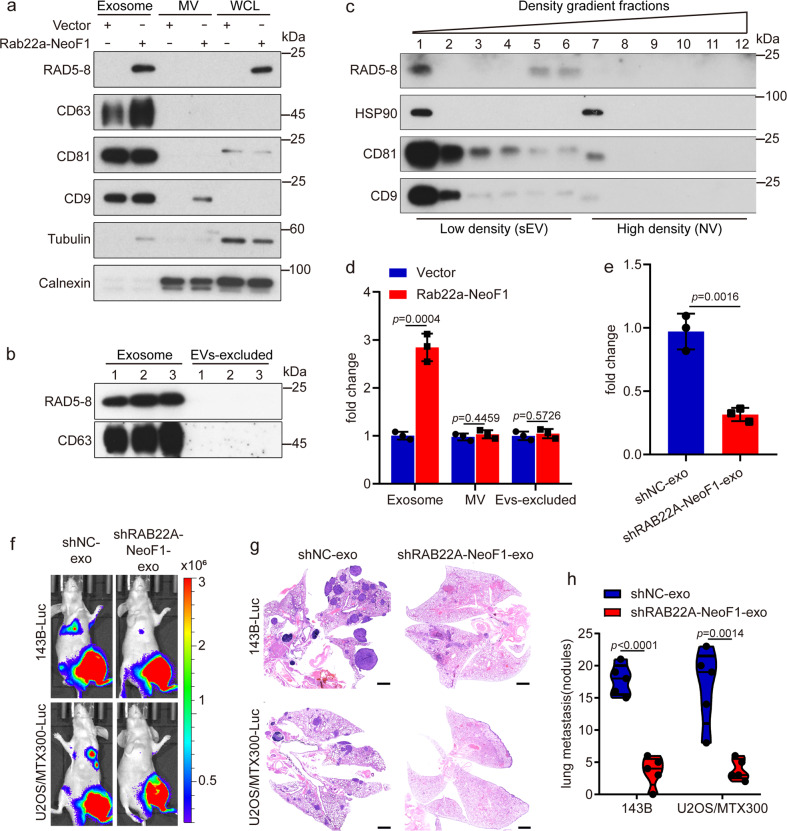
Rab22a-NeoF1 fusion protein is secreted into exosomes to promote metastasis. **a**, **b** The exosomes (Exosome), microvesicles (MV), whole-cell lysates (WCL), and EVs excluded supernatant (EVs-excluded) derived from the indicated stable 143B cells were prepared and analyzed by Western blotting. **c** High-resolution (12–36%) iodixanol gradients centrifugation was performed using the exosomes derived from ZOS-M cells as described in “Materials and methods” and followed by Western blotting. Data in **a**–**c** are representative of *n* = 3 biologically independent experiments. **d**, **e** 143B cells were incubated with exosomes (Exosome), microvesicles (MV), and EVs excluded supernatant (EVs-excluded) derived from the indicated stable 143B cells (**d**) or exosomes derived from the indicated stable ZOS-M cells (**e**) for 24 h. Cells were collected and subjected to the invasion assay. Data are mean ± s.d. of *n* = 3 biologically independent experiments. *P* values are shown. **f**–**h** Representative IVIS imaging (**f**), H&E-stained lung sections (**g**), and quantification of lung metastatic foci (**h**) from mice orthotopically injected with the 143B-Luc or U2OS/MTX300-Luc cells under treatment of exosomes derived from the indicated stable ZOS-M cells. *n* = 5 biologically independent mice. Data are mean ± s.d. *P* values are shown. Scale bar, 2 mm

Since exosomes containing bioactive molecules play crucial roles in the cell-to-cell communication,^7^ we reasoned that the secreted Rab22a-NeoF1 fusion protein may be present in exosomes. First, a small proportion of Rab22a-NeoF1 fusion proteins were co-localized with *cis*- or *trans*-Golgi (GM130 or TGN46), early endosomes (EEA1) and lysosomes (LAMP1), whereas there was a high Pearson correlation coefficient between Rab22a-NeoF1 and CD63, a marker of late endosome generally accepted as the precursors of exosomes (Supplementary Fig. 2a, b). Second, Rab22a-NeoF1 fusion protein was detected in the exosomes, but in neither the microvesicles (MV) nor the supernatants excluding EVs (EVs-excluded), isolated from the CM of 143B cells stably expressing Rab22a-NeoF1, but not Vector (Fig. 2a, b). These exosomes were validated by transmission electron microscopy and nanoparticle tracking analysis (Supplementary Fig. 2c, d). The presence of Rab22a-NeoF1 fusion protein in exosomes was further confirmed by the self-forming iodixanol gradient centrifugation at its endogenous and ectopic levels (Fig. 2c and Supplementary Fig. 2e, f). Third, the exosomal EGFP-labeled Rab22a-NeoF1 fusion protein, which was isolated from 143B cells stably expressing Rab22a-NeoF1-EGFP (Rab22a-NeoF1 conjugated with EGFP), could be taken up and internalized by U2OS cells after incubation for 15 min (Supplementary Fig. 2g, h). These results indicate that Rab22a-NeoF1 fusion protein is secreted into exosomes and could be endocytosed by its recipient cells.

To investigate whether the exosomal Rab22a-NeoF1 fusion protein is functional, we pre-treated 143B cells with exosomes derived from cells stably expressing Rab22a-NeoF1. As shown in Fig. 2d, the Rab22a-NeoF1 contained exosomes, neither MV nor EVs excluded supernatant, promoted cell invasion. Consistently, the promotion of cell invasion was significantly decreased in 143B cells treated with exosomes derived from ZOS-M cells stably knockdown *RAB22A*-NeoF1 compared to those derived from ZOS-M control cells (Fig. 2e). Furthermore, using the orthotopic osteosarcoma metastasis model in vivo, the enhancement of lung metastases was abolished in both 143B-Luc and U2OS/MTX300-Luc cells treated with exosomes derived from ZOS-M cells stably knockdown *RAB22A*-NeoF1 compared to those derived from ZOS-M control cells (Fig. 2f–h and Supplementary Fig. 2i). These results illustrate that Rab22a-NeoF1 fusion protein is present in exosomes to promote lung metastases of its negative cancer cells.

### Both secretion and function of exosomal Rab22a-NeoF1 fusion protein are dependent on its binding to HSP90

**Fig. 3:**
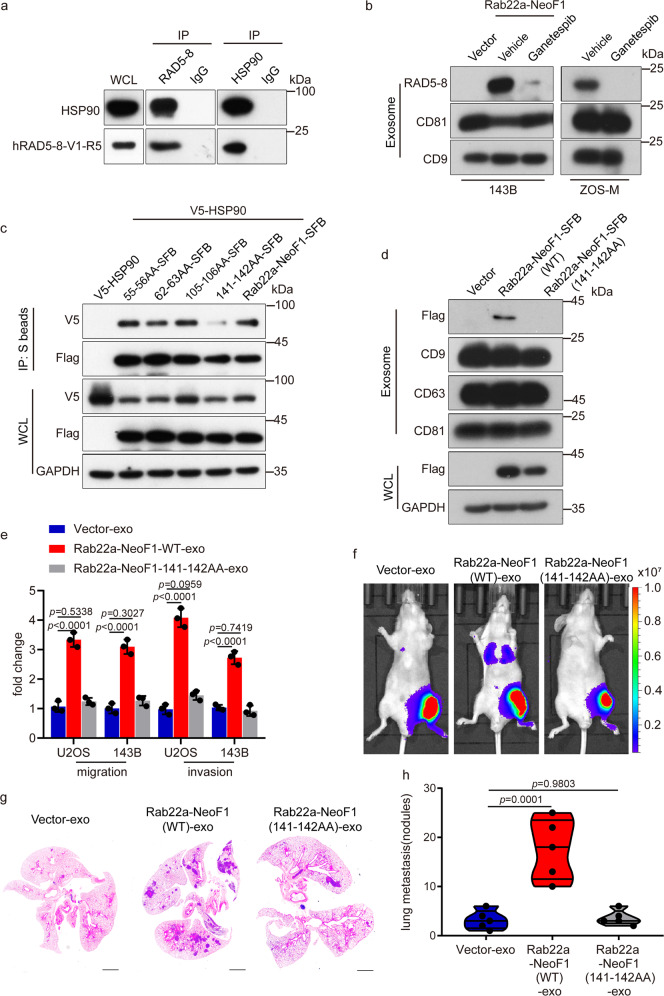
Both secretion and function of exosomal Rab22a-NeoF1 fusion protein are dependent on its binding to HSP90. **a** The co-IPs were performed using ZOS-M cells with anti-IgG, anti-mAb RAD5–8, or anti-HSP90 at their endogenous levels. WCL whole-cell lysate of ZOS-M cells. **b** The indicated stable 143B cells and ZOS-M cells were treated with the HSP90 inhibitor (Ganetespib) for 24 h, and then the exosomes were purified and analyzed by Western blotting. **c** The 293T cells were co-transfected with the indicated plasmids and then were lysed and analyzed by immunoprecipitation using S protein beads and Western blotting. **d** The Exosomes derived from the indicated stable 143B cells were isolated and analyzed by Western blotting. Data in **a**–**d** are representative of *n* = 3 biologically independent experiments. **e** The indicated cells were treated with exosomes derived from the indicated stable 143B cells for 24 h and then were subjected to migration and invasion assays. Data are mean ± s.d. of *n* = 3 biologically independent experiments. *P* values are shown. **f**–**h** Representative IVIS imaging (**f**), H&E-stained lung sections (**g**), and quantification of lung metastatic foci (**h**) from mice orthotopically injected 143B-Luc cells with exosomes derived from the indicated stable 143B cells. *n* = 5 biologically independent mice. Data are mean ± s.d. *P* values are shown. Scale bar, 2 mm

Next, we explored how Rab22a-NeoF1 fusion protein is secreted into exosomes. Using cells stably expressing Rab22a-NeoF1, we generated stable cells individually knocking down TSG101, ALIX, RAB11A, RAB27A, or RAB31A by shRNAs, the well-defined molecules related to the biogenesis, transport, and release of exosomes^11,13,27^ (Supplementary Fig. 3a, b). As shown in Supplementary Fig. 3c, Rab22a-NeoF1 fusion protein levels were decreased in exosomes from these stable cells, indicating that Rab22a-NeoF1 fusion protein is secreted into exosomes via the ESCRT-dependent pathway. Previous reports showed that cargos could be recognized by heat-shock proteins through its KFERQ-like motif for protein secretion;^28^ therefore, we analyzed the protein sequence of Rab22a-NeoF1 and found that there are four putative KFERQ-like motifs (Supplementary Fig. 3d), indicating that Rab22a-NeoF1 fusion protein may bind heat-shock proteins. In fact, numerous heat-shock proteins, including HSC70, HSP70, and HSP90, were shown in the list of TAP-MS data from the Rab22a-NeoF1 fusion protein interactome.^6^ These interactions between these heat-shock proteins and Rab22a-NeoF1 fusion protein in cells were further supported by co-immunoprecipitation (Fig. 3a and Supplementary Fig. 3e). To clarify whether the secretion of Rab22a-NeoF1 fusion protein into exosomes is dependent on heat-shock proteins, we treated cells with HSP90 inhibitor (Ganetespib), HSP70 and HSC70 inhibitors (VER15508 and Apoptozole), and found that inhibition of HSP90 dramatically decreased the exosomal Rab22a-NeoF1 fusion protein level from either ZOS-M cells harboring endogenous Rab22a-NeoF1 or 143B cells stably expressing Rab22a-NeoF1, compared to inhibition of HSP70 or HSC70 (Fig. 3b and Supplementary Fig. 3f). Interesting, we co-transfected HSP90 with the individual mutants of these KFERQ-like motifs in cells, and found that the 141-142AA mutant of Rab22a-NeoF1 almost lost its binding to HSP90 and completely abrogated its secretion into exosomes (Fig. 3c, d). Most importantly, the exosomes from cells stably expressing the 141-142AA mutant of Rab22a-NeoF1 lacked the enhancement of cell migration and invasion in both U2OS and 143B cells (Fig. 3e), as well as the lung metastases of 143B-Luc cells in the orthotopic osteosarcoma metastasis model in vivo (Fig. 3f–h and Supplementary Fig. 3g). These results reinforce that Rab22a-NeoF1 fusion protein is sorted into exosomes by binding to HSP90 via one of its KFERQ-like motifs (RVLFLN^142^) to promote lung metastases of its negative cancer cells.

### The functions of the exosomal Rab22a-NeoF1 on recipient cells are dependent on PYK2 from donor cells

**Fig. 4:**
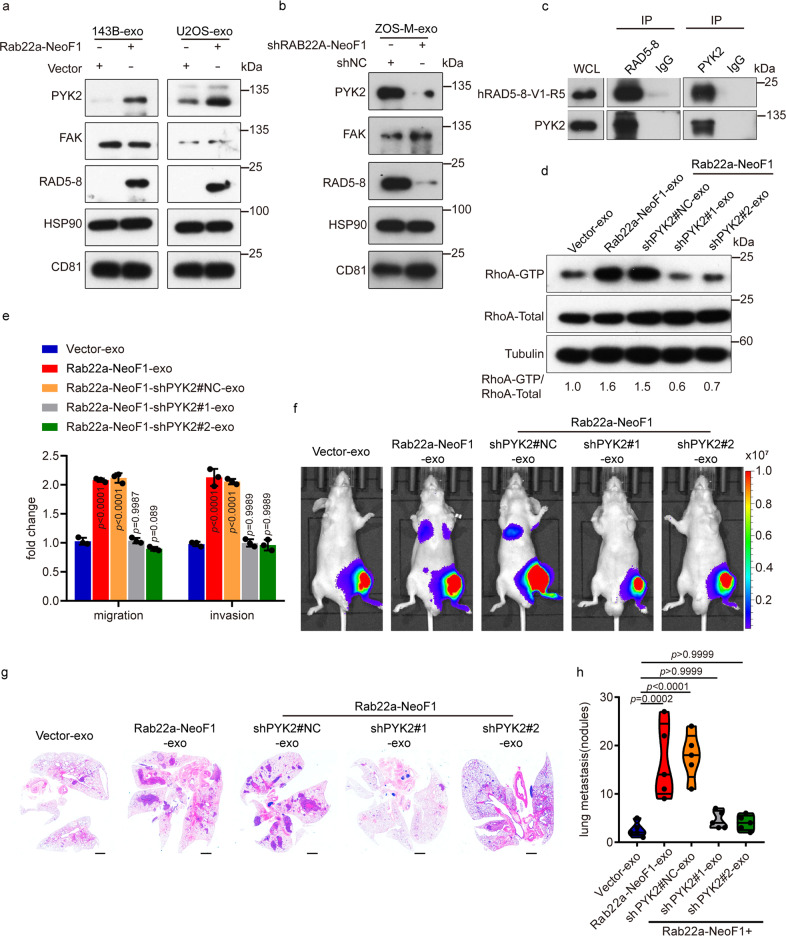
The functions of exosomes containing Rab22a-NeoF1 fusion protein on its negative recipient cancer cells requires its binding partner PYK2 from donor cells. **a**, **b** The exosomes derived from the indicated stable 143B and U2OS (**a**), as well as ZOS-M (**b**) cells were purified and subjected to Western blotting. **c** The co-IPs were performed using ZOS-M cells with anti-mAb RAD5–8, anti-PYK2, or anti-IgG at their endogenous levels. WCL whole-cell lysate of ZOS-M cells. **d** The 143B cells were treated with the indicated exosomes for 1 h and then were lysed. Rho-GTP forms were pulldown by RBD beads and were subjected to Western blotting. The relative intensity of RhoA-GTP was quantified with the ImageJ software and normalized with RhoA-Total. Data in **a**–**d** are representative of *n* = 3 biologically independent experiments. **e** The 143B cells were treated with the indicated exosomes for 24 h and then were subjected to migration and invasion assays. Data are mean ± s.d. of *n* = 3 biologically independent experiments. *P* values are shown. **f**–**h** Representative IVIS imaging (**f**), H&E-stained lung sections (**g**), and quantification of lung metastatic foci (**h**) from mice orthotopically injected 143B-Luc cells with the indicated exosomes. *n* = 5 biologically independent mice. Data are mean ± s.d. *P* values are shown. Scale bar, 2 mm

To explore the mechanism by which the exosomal Rab22a-NeoF1 fusion protein promotes metastasis, quantitative mass spectrometry of exosomes derived from 143B cells stably expressing Rab22a-NeoF1 (Rab22a-NeoF1-exo) or Vector (Vector-exo) were performed, and 173 among over 2000 proteins were differently expressed in these two groups of exosomes (Supplementary Fig. 4a, b). Notably, PYK2, sharing similar domain organization with focal adhesion kinase (FAK), that has been reported to activate RhoA,^29^ was much higher in Rab22a-NeoF1-exo compared to Vector-exo, which was validated by Western blotting (Fig. 4a). In contrast, PYK2 was decreased in exosomes derived from ZOS-M cells stably knocking down *RAB22A*-NeoF1 compared to those derived from ZOS-M control cells (Fig. 4b). Moreover, the interaction between Rab22a-NeoF1 and PYK2, but not FAK, was detected by co-immunoprecipitation in cells at their endogenous and ectopic levels (Fig. 4c and Supplementary Fig. 4c). More importantly, activation of RhoA was observed in 143B cells treated with Rab22a-NeoF1-exo, but not Vector-exo (Supplementary Fig. 4d), and the promotion of cell migration and invasion by Rab22a-NeoF1-exo was abolished in both U2OS and 143B cells pre-incubated with CT04, a RhoA-specific inhibitor (Supplementary Fig. 4e), indicating that exosomes containing Rab22a-NeoF1 promote cell migration and invasion of their recipient cells through activation of RhoA. Next, we purified the exosomes derived from 143B stable cells simultaneously knocking down PYK2 with shRNA and expressing Rab22a-NeoF1 (Rab22a-NeoF1-shPYK2#1-exo and Rab22a-NeoF1-shPYK2#2-exo) and those derived from 143B cells simultaneously expressing Rab22a-NeoF1 and non-targeting shRNA (Rab22a-NeoF1-shPYK2#NC-exo) as the control (Supplementary Fig. 4f). Strikingly, the enhancement of RhoA-GTP (Fig. 4d), cell migration, and invasion in 143B cells in vitro (Fig. 4e), as well as lung metastases of 143B-Luc cells in the orthotopic osteosarcoma metastasis model in vivo (Fig. 4f–h and Supplementary Fig. 4g) were observed by either Rab22a-NeoF1-exo or Rab22a-NeoF1-shPYK2#NC-exo, but not by either Rab22a-NeoF1-shPYK2#1-exo or Rab22a-NeoF1-shPYK2#2-exo compared to Vector-exo. These results determine that the exosomal Rab22a-NeoF1 fusion protein promotes cell migration, invasion, and metastasis of its negative recipient cells via activation of RhoA by PYK2 from donor cells in osteosarcoma.

### The exosomal Rab22a-NeoF1 promotes pre-metastatic niche formation via enhancing bone marrow-derived macrophages recruitment

**Fig. 5:**
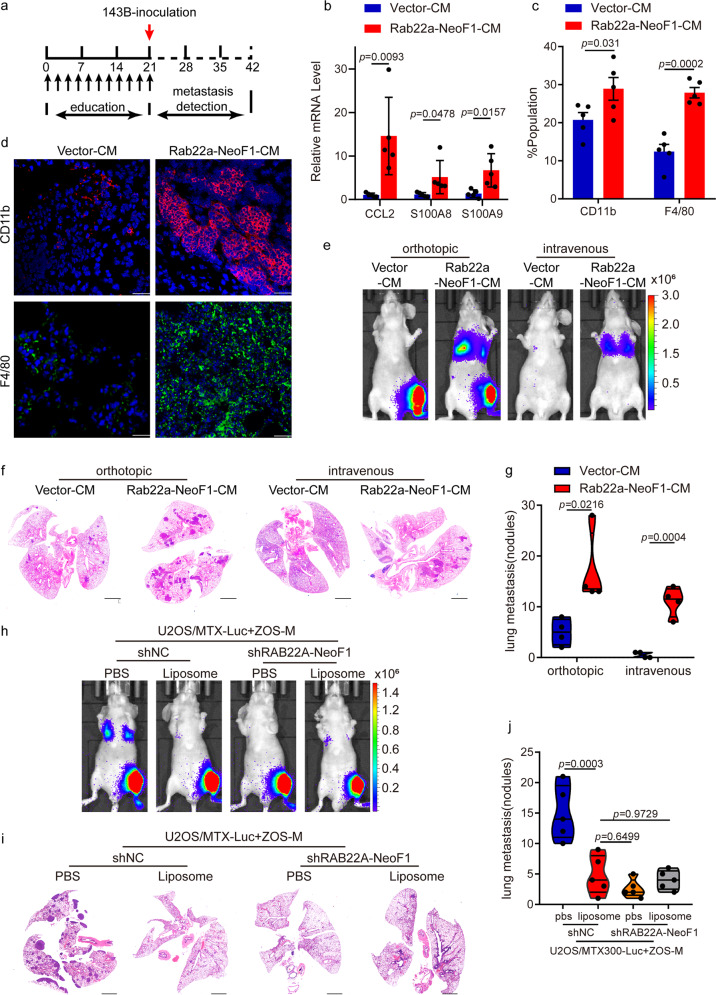
The functions of exosomal Rab22a-NeoF1 fusion protein requires recruiting BMDMs to the pulmonary pre-metastatic niche. **a** Schematic illustration for pre-educated tumor-free mice model. **b** The indicated gene mRNA levels from the lungs of mice treated with the indicated conditioned media (CM) were quantified by qPCR. *n* = 5 biologically independent mice. Data are mean ± s.d. *P* values are shown. **c** Mice lungs pre-educated with the indicated conditioned media (CM) were homogenized and analyzed by flow cytometry for numbers of both CD11b+ cells and F4/80+ cells. *n* = 5 biologically independent mice. Data are mean ± s.d. *P* values are shown. **d** Representative immunofluorescent analyses of CD11b and F4/80 expression in the lungs from mice treated with the indicated conditioned media (CM). Scale bar, 100 μm. **e**–**g** Mice were pre-educated with the indicated conditioned media (CM) for 3 weeks, and then were orthotopically or tail vein injected with 143B-Luc cells for the indicated periods. The lung metastases were analyzed. Representative IVIS imaging (**e**), H&E-stained lung sections (**f**), and quantification of lung metastatic foci (**g**). *n* = 4 biologically independent mice. Data are mean ± s.d. *P* values are shown. Scale bar, 2 mm. **h**–**j** Mice orthotopically co-injected U2OS/MTX300-Luc cells with either ZOS-M-shNC cells or ZOS-M-shRAB22A-NeoF1 cells at the 10:1 ratio, and then intravenously injected liposome or PBS twice a week for 3 weeks and the lung metastases were analyzed. Representative IVIS imaging (**h**), H&E-stained lung sections (**i**), and quantification of lung metastatic foci (**j**). *n* = 5 biologically independent mice. Data are mean ± s.d. *P* values are shown. Scale bar, 2 mm

It is well known that cancer cells secrete factors contributing to the formation of pre-metastatic niche by stimulating the bone marrow-derived cells (BMDCs) mobilization or inducing the extracellular matrix remodeling.^14,23,24,30^ To investigate the role of exosomal Rab22a-NeoF1 fusion protein in the formation of pre-metastatic niche, we pre-educated tumor-free mice by injection of the CM, as illustrated in Fig. 5a. Interestingly, higher expressions of CCL2, S100A8, and S100A9, molecular markers promoting pre-metastatic niche formation, were induced in lungs by Rab22a-NeoF1-CM compared to those by Vector-CM (Fig. 5b). Mice injected with Rab22a-NeoF1-CM had higher pulmonary numbers of both CD11b+ myeloid cells and F4/80+ macrophages compared to those injected with Vector-CM (Fig. 5c). This was further supported by the CD11b and F4/80 staining in lungs of these mice (Fig. 5d). Furthermore, using the orthotopic osteosarcoma metastasis model in vivo and the tail vein injection metastasis model in vivo, the lung metastases of 143B-Luc cells were significantly increased in mice pre-educated by Rab22a-NeoF1-CM compared to those pre-educated by Vector-CM (Fig. 5e–g and Supplementary Fig. 5a).

To determine whether bone marrow-derived macrophages (BMDMs) were responsible for such a promotion of lung metastasis, the liposome-mediated macrophage depletion mouse model was used.^31^ We orthotopically co-injected U2OS/MTX300-Luc cells with ZOS-M-sh*RAB22A*-NeoF1 cells or ZOS-M-shNC cells at the 10:1 ratio into mice treated with liposomal clodronate (liposome) or phosphate-buffered saline (PBS), and found that liposome treatment significantly decreased the lung metastases of U2OS/MTX300-Luc cells in mice bearing tumors from their mixtures with ZOS-M-shNC cells, but not with ZOS-M-sh*RAB22A*-NeoF1 cells (Fig. 5h–j and Supplementary Fig. 5b). Furthermore, liposome treatment also significantly decreased the lung metastases of 143B-Luc cells in mice pre-educated by Rab22a-NeoF1-exo, but not by Vector-exo, using the orthotopic osteosarcoma metastasis model in vivo (Supplementary Fig. 5c–f). These results illustrate that BMDMs are required for Rab22a-NeoF1 fusion protein to promote its negative recipient cancer cells to metastasize to lungs in osteosarcoma.

### The exosomal Rab22a-NeoF1 promotes M2 macrophage polarization through its binding partner PYK2 from donor cells

**Fig. 6:**
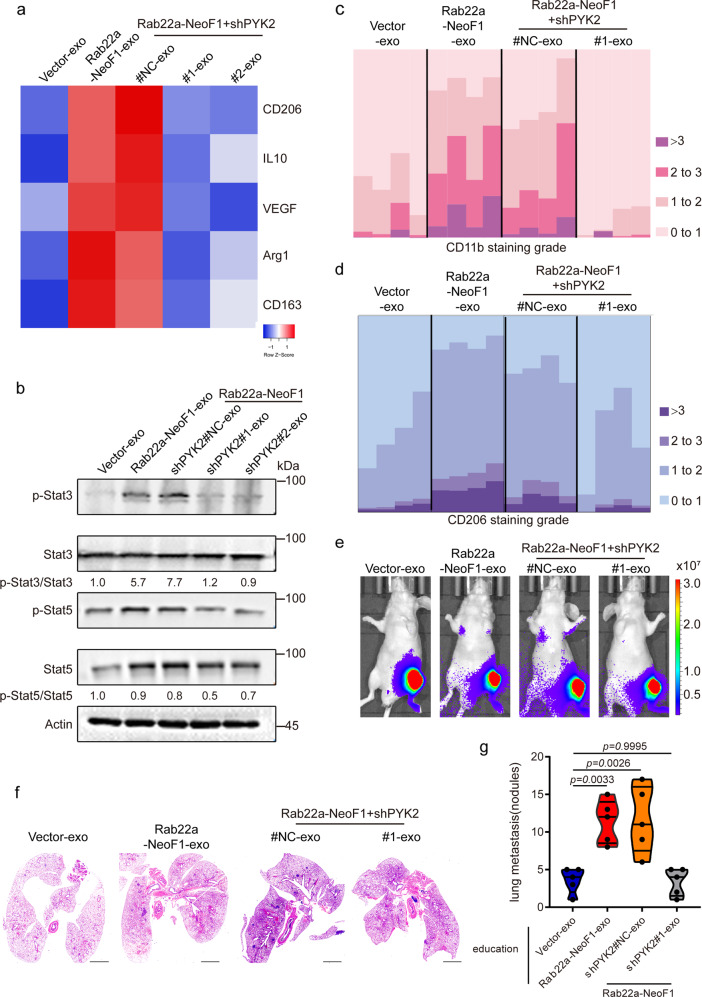
The exosomal Rab22a-NeoF1 fusion protein promotes M2 polarization in its recipient macrophages via its binding partner PYK2. **a** Raw264.7 cells were incubated with the indicated exosomes, and differential expression patterns of the M2-like macrophage markers were presented by a heatmap. **b** Raw264.7 cells treated with the indicated exosomes and then were subjected to Western blotting. The relative intensity of p-Stat3 and p-Stat5 were quantified with the ImageJ software and normalized with Stat3-Total and Stat5, respectively. Data are representative of *n* = 3 biologically independent experiments. **c**, **d** Mice lungs pre-educated with the indicated exosomes were isolated and analyzed by IHC for expression CD11b (**c**) and CD206 (**d**) expression. *n* = 4 biologically independent mice. Bar plots show CD11b and CD206 staining grade. **e**–**g** Mice were pre-educated with the indicated exosomes for 3 weeks, and then were orthotopically injected with 143B-Luc cells. The lung metastases were analyzed after 3 weeks. Representative IVIS imaging (**e**), H&E-stained lung sections (**f**), and quantification of lung metastatic foci (**g**). *n* = 5 biologically independent mice. Data are mean ± s.d. *P* values are shown. Scale bar, 2 mm

There are two major phenotypes of macrophage polarization (M1 and M2) in tumors. Typically, the M1 type is thought to inhibit tumor growth, while M2 polarization has a supportive role to promote tumor progression.^32^ To look into how BMDMs are affected by the exosomal Rab22a-NeoF1 fusion protein during lung metastasis, we hypothesized that the exosomal Rab22a-NeoF1 fusion protein may affect macrophage polarization. To this end, Raw246.7 and THP-1 cells, two commonly used macrophages, were treated with Rab22a-NeoF1-exo or Vector-exo. As shown in Supplementary Fig. 6a, b, the mRNA levels of M2 polarization markers (e.g., CD206 and CD163), but not M1-type markers, were dramatically increased by the exosomal Rab22a-NeoF1 fusion protein in both Raw246.7 and THP-1 cells. Furthermore, the promotions of exosomal Rab22a-NeoF1 fusion protein on the M2 polarization markers (Fig. 6a and Supplementary Fig. 6c) were diminished in Raw246.7 or THP-1 cells treated with either Rab22a-NeoF1-shPYK2#1-exo or Rab22a-NeoF1-shPYK2#2-exo. It is well known that signal transducer and activator of transcription 3 (Stat3) activation plays a key role in polarization of M2 macrophage,^33^ and PYK2 forms a positive feedback loop with Stat3 to promote cancer metastasis.^34^ Indeed, activation of Stat3, but not Stat5, and cell migration were significantly increased in Raw246.7 cells treated with Rab22a-NeoF1-exo compared to those treated by Vector-exo (Fig. 6b and Supplementary Fig. 6d), and such increases were abolished when Raw246.7 cells were treated with either Rab22a-NeoF1-shPYK2#1-exo or Rab22a-NeoF1-shPYK2#2-exo. This indicates that the exosomal PYK2 as a binding partner of Rab22a-NeoF1 is required for activation of Stat3 in its recipient macrophages. Consistent with this, Stat3 inhibitors, such as Niclosamide and SH-4-54, could diminish the M2 polarization markers in Raw246.7 cells treated with exosomes containing Rab22a-NeoF1 (Supplementary Fig. 6e). More importantly, the CD11b+ myeloid cells and the M2 polarization markers (CD206+) in pulmonary pre-metastatic niche (Fig. 6c, d and Supplementary Fig. 6f, g), as well as of lung metastases of 143B-Luc cells (Fig. 6e–g and Supplementary Fig. 6h), were significantly reduced from the mice pre-educated with Rab22a-NeoF1-shPYK2#1-exo compared to those with Rab22a-NeoF1-shPYK2#NC-exo. These results demonstrate that the exosomal Rab22a-NeoF1 fusion protein promotes M2 polarization to induce pulmonary pre-metastatic niche formation and subsequently facilitate lung metastases of its negative recipient cancer cells in osteosarcoma, which is dependent on its binding partner PYK2 from donor cells.

### Disrupting the interaction between Rab22a-NeoF1 and PYK2 by the 1–10 amino acid (a.a.)-iRGD peptide prevents the recipient osteosarcoma cells to metastasize to lungs

**Fig. 7:**
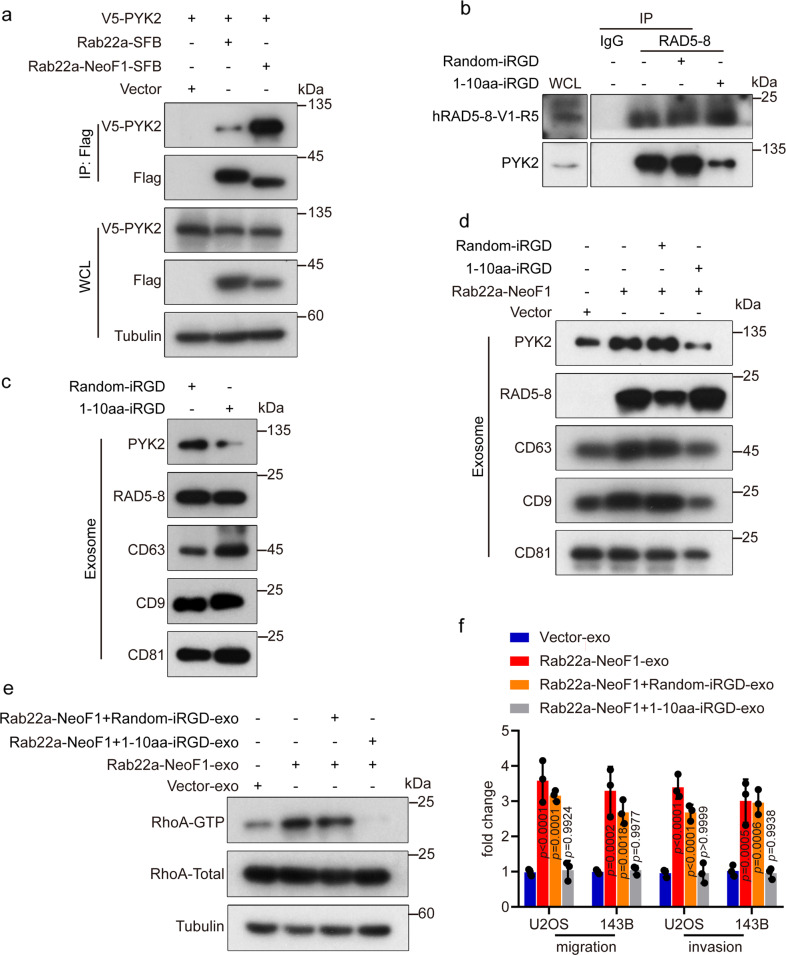
Blockage of the interaction between Rab22a-NeoF1 and PYK2 inhibits the RhoA activation, migration, and invasion of the recipient cells induced by the exosomal Rab22a-NeoF1 fusion protein. **a** The 293T cells were co-transfected with the indicated plasmids and then were lysed and analyzed by immunoprecipitation using anti-Flag beads followed by Western blotting. **b** ZOS-M cells were incubated with the indicated peptide for 24 h, and then were lysed and subject to immunoprecipitation using anti-IgG or anti-mAb RAD5–8, followed by Western blotting. WCL whole-cell lysate of ZOS-M cells. **c**, **d** ZOS-M cells (**c**) and the indicated stable 143B cells (**d**) were treated with the indicated peptide for 24 h, and then the exosomes were purified and analyzed by Western blotting. **e** U2OS cells were incubated with the indicated exosomes for 1 h and then were lysed and subjected to RhoA activation assay. Data in **a**–**e** are representative of *n* = 3 biologically independent experiments. **f** U2OS and 143B cells were treated with the indicated exosomes for 24 h, and then were subjected to migration and invasion assays. Data are mean ± s.d. of *n* = 3 biologically independent experiments. *P* values are shown

Next, we sought to map the region of Rab22a-NeoF1 fusion protein responsible for its binding to PYK2. As shown in Fig. 7a and Supplementary Fig. 7a, b, Rab22a-NeoF1 fusion protein had a much stronger binding affinity to PYK2 compared to wild-type Rab22a, and the 1–10 a.a. of Rab22a-NeoF1 was responsible for its binding to PYK2. These results were very interesting, as Rab22a-NeoF1 fusion protein has a higher binding affinity to SmgGDS607 via the 1–10 a.a. compared to wild-type Rab22a.^6^

**Fig. 8:**
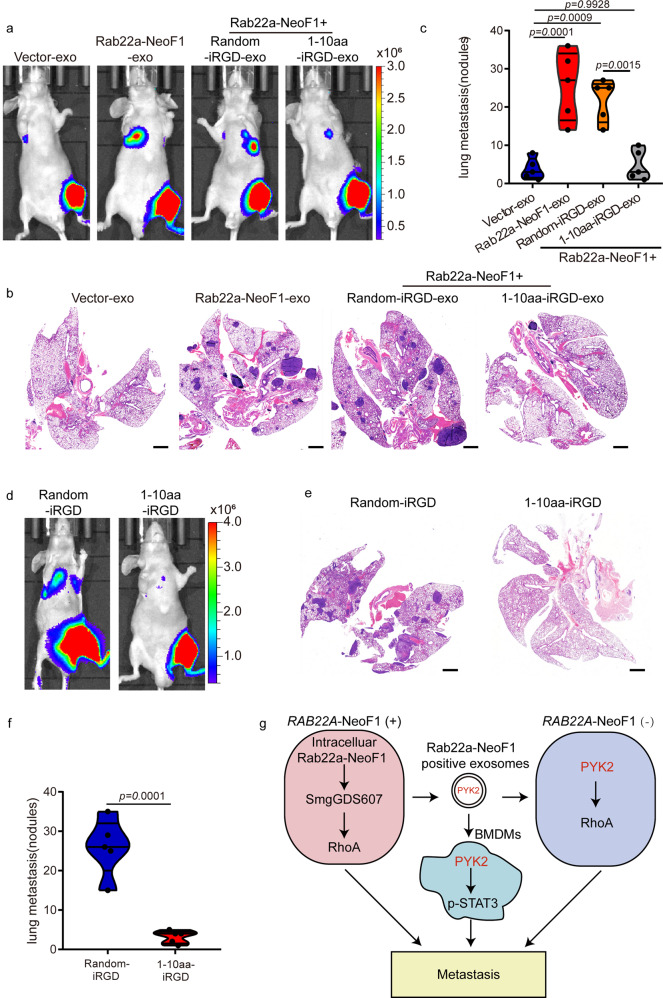
Blockage of the interaction between Rab22a-NeoF1 and PYK2 inhibits lung metastases of the recipient cells induced by the exosomal Rab22a-NeoF1 fusion protein. **a**–**c** Representative IVIS imaging (**a**), H&E-stained lung sections (**b**), and quantification of lung metastatic foci (**c**) from mice orthotopically injected 143B-Luc cells, and then were treated with the indicated exosomes. *n* = 5 biologically independent mice. Data are mean ± s.d. *P* values are shown. Scale bar, 2 mm. **d**–**f** Representative IVIS imaging (**d**), H&E-stained lung sections (**e**), and quantification of lung metastatic foci (**f**) from mice orthotopically co-injected U2OS/MTX300-Luc cells with ZOS-M cells at the 10:1 ratio under the treatment of either 1–10 a.a.-iRGD or random-iRGD peptide. *n* = 5 biologically independent mice. Data are mean ± s.d. *P* values are shown. Scale bar, 2 mm. **g** The proposed model for function and mechanism of Rab22a-NeoF1 fusion protein in osteosarcoma. A small proportion of tumor cells positive for Rab22a-NeoF1 fusion protein promote lung metastasis at least by three ways, Rab22a-NeoF1 fusion protein activates the cellular RhoA by constitutively binding to SmgGDS607; the exosomal Rab22a-NeoF1 fusion protein promotes RhoA activation in its negative recipient cancer cells by the exosomal PYK2 from donor cells, as well as facilitates the pre-metastatic niche formation in the lung by promoting BMDMs recruitment and by increasing M2 macrophages via the exosomal PYK2-mediated Stat3 activation from donor cells

Since the 1–10-a.a.-internalizing RGD (iRGD) peptide, previously called iRGD-WT, has been tested to be properly worked using osteosarcoma cells in vitro and in vivo,^6^ we synthesized the random-iRGD peptide, containing the same 1–10 a.a. with a randomized sequence, as its negative control, to test their effects on the functions of the exosomal Rab22a-NeoF1 fusion protein. First, the interaction between Rab22a-NeoF1 fusion protein and PYK2 at their endogenous and ectopic levels were dramatically diminished in cells treated with the 1–10 a.a.-iRGD, but not the random-iRGD (Fig. 7b and Supplementary Fig. 7c, d). Second, the 1–10 a.a.-iRGD, but not the random-iRGD, abolished the enhancement of the secretion of exosomal PYK2 from either 143B cells stably expressing Rab22a-NeoF1 or ZOS-M cells harboring endogenous Rab22a-NeoF1 (Fig. 7c, d). Third, we purified the exosomes derived from 143B cells stably expressing Rab22a-NeoF1 under the treatment of either 1–10 a.a.-iRGD (Rab22a-NeoF1 + 1–10 a.a.-iRGD-exo) or random-iRGD (Rab22a-NeoF1 + random-iRGD-exo) for 24 h, and found that the promotion of RhoA activation, cell migration, and invasion in 143B cells in vitro (Fig. 7e, f and Supplementary Fig. 7e), as well as lung metastases of 143B-Luc cells in the orthotopic osteosarcoma metastasis model in vivo were observed by both Rab22a-NeoF1-exo and Rab22a-NeoF1+random-iRGD-exo, but not by Rab22a-NeoF1+1–10 a.a.-iRGD-exo, compared to Vector-exo (Fig. 8a–c and Supplementary Fig. 7f). Fourth, by orthotopically co-injecting U2OS/MTX300-Luc cells with ZOS-M cells at the 10:1 ratio into mice, their lung metastases of U2OS/MTX300-Luc cells were significantly decreased by treating with the 1–10 a.a.-iRGD, but not with the random-iRGD, (Fig. 8d–f and Supplementary Fig. 7g). Taken together, our results demonstrate that perturbation of the interaction between Rab22a-NeoF1 and PYK2 using the 1–10 a.a.-iRGD peptide abrogates the promotion of its negative recipient cancer cells metastasizing to lungs induced by Rab22a-NeoF1 fusion protein.

## Discussion

The fusion genes act as oncogenic driver in numerous human cancers.^35,36^ Exosome derived from tumor cells has been linked to tumor initiation and progression.^37^ In this report, we demonstrate for the first time that osteosarcoma cells harboring *RAB22A*-NeoF1 fusion gene, a driver of metastasis, may secrete the Rab22a-NeoF1 enclosed exosomes into tumor microenvironment to affect the functions of their surrounding recipient cells, such as macrophages and tumor cells negative for *RAB22A*-NeoF1.

The sorting of proteins into exosomes is mediated by a variety of pathways, most of which are still not fully elucidated.^27^ It has been recently reported that heat-shock proteins, such as HSC70 and HSP90, participate in the process of unconventional protein secretion through binding to their client proteins that contain KFERQ-like motifs.^28,38^ As reported here, Rab22a-NeoF1 fusion protein is sorted into exosomes through binding to heat-shock proteins (e.g., HSP90) via one of its KFERQ-like motifs (RVLFLN^142^), and secreted by the ESCRT-dependent pathway (Fig. 3a–d and Supplementary Fig. 3a–f). Notably, there is also a KFERQ-like motif in Rab22a-NeoF5, but not in Rab22a-NeoF2, 3, 4, 6. Similar to Rab22a-NeoF1, Rab22a-NeoF5 fusion protein, but not other Rab22a-NeoFs fusion proteins, was also detectable in exosomes (Supplementary Fig. 8a, b). The secretion of Rab22a-NeoF1, 5 fusion proteins into exosomes, which may prevent them from degradation by proteasome/lysosome, may explain why the Rab22a-NeoF1, 5 fusion proteins were relatively stable in cells compared to Rab22a-NeoF2, 3, 4, 6 fusion proteins, as shown recently by our group.^6^

In tumors, cancer cells generally promote the M2 phenotype macrophage polarization to remodel tumor microenvironment and to facilitate tumor progression.^39^ For instance, the secreted LOX by tumor cells^40^ or exosomal MIF^23^ is able to create the favorable microenvironment in the distant organ for subsequent metastasis. As reported here, the exosomal Rab22a-NeoF1 fusion protein may enter the circulation to promote pulmonary pre-metastatic niche formation via recruiting BMDMs and inducing M2 phenotype macrophage polarization. This function may be required for the activation of Stat3 in its recipient macrophages by the exosomal PYK2, a binding partner of Rab22a-NeoF1 fusion protein from its positive donor cells (Figs. 5, 6 and Supplementary Figs. 5, 6). Our findings are consistent with the literature, showing that Stat3 activation is critical for the polarization of M2 macrophage,^33^ and that PYK2 promotes cancer metastasis by forming a positive feedback loop with Stat3.^34^ Therefore, it is possible that cancer progression may be more complicated once some cancer cells are positive for any fusion protein, as the fusion protein may have multiple ways to accelerate cancer progression and subsequently accounts for patient outcome.

PYK2 via binding to Rab22a-NeoF1 fusion protein is also enriched in exosomes, and the exosomal PYK2 induces RhoA activation in its recipient cancer cells negative for Rab22a-NeoF1 to promote migration, invasion, and lung metastases of these cancer cells (Fig. 4d–h and Supplementary Fig. 4d–g). On the other hand, the intercellular activation of RhoA mediated by Rab22a-NeoF1 fusion protein in its positive cancer cells is achieved via its constitutively binding to SmgGDS607 to release RhoA to become active, as shown recently by our group.^6^ *RAB22A*-NeoF1 fusion gene is expressed in cells, its protein is high enough to constitutively bind SmgGDS607, a specific noncanonical GEF protein for RhoA and RhoC,^41^ to facilitate the activation of RhoA. This phenomenon seems to be very striking, Rab22a-NeoF1 fusion protein may be limited in its negative recipient cancer cells as it is taken up by exosome endocytosis, and then it binds to PYK2 kinase that has a high efficiency to activate RhoA. In other words, Rab22a-NeoF1 fusion protein activates RhoA via two distinct ways dependent on its availability.

Notably, the interaction of Rab22a-NeoF1 with PYK2, not with smgGDS607, was blocked in cells by inhibiting HSP90 with either Tanespimycin or Ganetespib (Supplementary Fig. 9). Therefore, we speculate that Rab22a-NeoF1 fusion protein may have two different pools in cells, one for binding to smgGDS607 to activate intercellular RhoA, and another one for binding to both HSP90 and PYK2 to be sorted into exosomes. The exosomal Rab22a-NeoF1 fusion protein functions in its negative recipient cells, such as activation of RhoA and Stat3 in cancer cells and macrophages, respectively, which is dependent on its binding partner PYK2 from its positive donor cells. This proposal may be illustrated as Fig. 8g, perfectly explaining that a small proportion of osteosarcoma cells positive for Rab22a-NeoF1 promote lung metastases of the entire cancer cells, as recently reported in one of the patients from our group.^6^

We have generated the 1–10 a.a.-iRGD peptide, 1–10 a.a. of Rab22a-NeoF1 conjugated to the cell-penetrating peptide iRGD, which was able to prevent lung metastasis of cancer cells positive for *RAB22A*-NeoF1 in the orthotopic osteosarcoma metastasis model.^6^ Notably, the 1–10 a.a. of Rab22a-NeoF1 was also responsible for the interaction of Rab22a-NeoF1 with PYK2, and the 1–10 a.a.-iRGD peptide abolished the exosomal Rab22a-NeoF1-mediated lung metastases of its negative recipient cells using the orthotopic osteosarcoma metastasis model (Fig. 8a–f). Therefore, the 1–10 a.a.-iRGD peptide disturbs the Rab22a-NeoF1 binding to both SmgGDS607 and PYK2, and then would in principle diminish lung metastasis in cancer patients who are positive for *RAB22A*-NeoF1 fusion gene. Our findings provide a promising therapeutic strategy to prevent metastasis in a subset of cancer patients who are positive for the Rab22a^1–38^ fusion gene(s), as the Rab22a^1–38^ portion governs the metastasis-promoting function of Rab22a-NeoFs.

## Materials and methods

### Cell lines and cell culture

The human cell lines 293T, THP-1, U2OS, 143B and mouse cell line Raw246.7 were purchased from ATCC. The U2OS/MTX300 and ZOS-M cell lines were described previously.^42^ The 143B-Luc and U2OS/MTX300-Luc cells expressing luciferase were cultured as described previously.^43^ All cell lines were cultured in Dulbecco’s modified Eagle’s medium (DMEM) (Invitrogen) supplemented with 10% (vol/vol) fetal bovine serum (FBS) (Gibco, USA) and 1% (vol/vol) penicillin and streptomycin (Beyotime Biotech, China). All cell lines used in this research were authenticated by using short-tandem repeat profiling >6 months ago when this project was initiated and were cultured no >2 months.

To analyze the function of CM or exosomes containing Rab22a-NeoF1 fusion protein, U2OS, 143B, and Raw246.7 cells were incubated with the indicated stable cell-derived CM (100 μL) or exosomes (2 μg/mL) for 24 h and then were subjected to migration and invasion assays.

To assess which phenotype of macrophages was induced by exosomes containing Rab22a-NeoF1, Raw246.7, and THP-1 cells were treated with exosomes (10 μg) derived from the indicated stable cells for 24 h and then were lysed in TRIzol (Thermo Fisher) for M2 marker expression analysis.

### Plasmid construction and lentivirus production

Complementary DNAs (cDNAs) for HSP70, HSP90, HSC70, FAK, and PYK2 were amplified by PCR and cloned into the pcDNA3.1 vector with V5 tag. cDNAs for smgGDS607 was amplified by PCR and cloned into the pcDNA3.1 vector with HA tag. *RAB22A*-NeoF1 was amplified by PCR and cloned into the C-SFB vector. *RAB22A*-NeoF1-AA mutant (L140A + N141A) was conducted by Mut Express® II Fast Mutagenesis Kit (Vazyme) and verified by DNA-sequencing. shRNAs against human RAB11A, human RAB27A, human RAB35A, human ALIX, human TSG101, human PYK2, and human Rab22a-NeoF1 was inserted into PLKO.1 vector (Sigma). The shRNAs used are listed:

RAB22A-NeoF1 shRNA: AATCCAGATTCAAGTATCTACCCAA

PYK2 shRNA#1: CCCAGGAGAGAATGAAGCAAA

PYK2 shRNA#2: GATGTTGGTTTAAAGCGATTT

RAB11A shRNA: AGTTGTCCTTATTGGAGATTC

RAB27A shRNA: CGGATCAGTTAAGTGAAGAAA

RAB31A shRNA: TGATGATGTGTGCCGAATATT

ALIX shRNA: CCAGAACAAATGCAGTGATAT

TSG101 shRNA: GCCTTATAGAGGTAATACATA

The Rab22a-NeoF1 and Rab22a-NeoF1-141-142AA mutant was cloned into the PSIN-EF2-Puro lentiviral vector (Invitrogen). The stable overexpression or knockdown cells were generated by transduction with lentivirus. The viral particles were produced in 293T cells. Infected cells were selected by puromycin for 7 days and confirmed at the protein level.

### CM preparation

For CM collection experiments, 2.5 × 10^6^ Rab22a-NeoF1 or Vector 143B cells were plated on 15-cm dishes in DMEM supplemented with 10% FBS and allowed to adhere overnight. The media were then replaced by DMEM supplemented without FBS. Two days later, the CM was collected, centrifuged at 300 × *g* for 10 min, filtered, and aliquots were stored at −80 °C. The same procedure was followed for collection of CM from 4 × 10^6^ shNC or shRAB22A-NeoF1 ZOS-M cells.

### EV isolation

EVs were isolated by differential ultracentrifugation as described previously.^44^ EV-depleted FBS was prepared by overnight ultracentrifugation at 100,000 × *g*. Osteosarcoma cells were washed by PBS twice and cultured in DMEM supplemented with 10% EV-depleted FBS. Supernatants derived from cells were collected after 48 h and subjected to successive centrifugation. In brief, supernatants were centrifuged at 300 × *g* for 10 min at 4 °C to pellet floating cells. Supernatants were centrifuged at 2000 × *g* for 20 min at 4 °C to pellet dead cells. Supernatants were centrifuged at 10,000 × *g* for 40 min at 4 °C to pellet MVs. Supernatants were centrifuged at 100,000 × *g* for 90 min at 4 °C. After centrifugation, the supernatants (EVs-excluded) were collected, aliquots were stored at −80 °C, and the pelleted exosomes (Exosome) were resuspended in PBS and collected by ultracentrifugation at 100,000 × *g* for 90 min.

### Iodixanol density gradient fractionation

For iodixanol density gradient separation, exosomes (100 K pellet) were overlaid on top of the discontinuous iodixanol gradient. The gradients are made by diluting a 60% iodixanol with 0.25 M sucrose, 10 mM Tris-HCl, pH 7.5 (12–36% or 5–40%). Next, this mixture solution was centrifuged at 100,000 × *g* for 18 h at 4 °C and 12 individual 1 mL fractions were collected. The exosomes were pelleted by ultracentrifugation at 100,000 × *g* for 90 min at 4 °C.

### Western blotting and co-immunoprecipitation

Briefly, whole-cell lysates were prepared by using RIPA buffer (150 mM NaCl, 5 mM EDTA, 50 mM Tris-HCl, 0.5% NP-40) supplemented with phosphatase inhibitor and protease inhibitor cocktails (Thermo Scientific). The cell lysates were incubated with anti-S protein beads, anti-Flag beads, or anti-V5 beads (Sigma) overnight at 4 °C, and then the beads were washed six times with cold RIPA buffer and eluted with 5× SDS sample buffer. The immunoprecipitates were separated by sodium dodecyl sulfate–polyacrylamide gel electrophoresis and transferred onto polyvinylidene difluoride membranes (Millipore). The membranes were blocked with 5% non-fat milk for 1 h at room temperature and incubated with primary antibodies and horseradish peroxidase-conjugated secondary antibody. The clarity ECL substrate (Pierce, USA) or high-sig ECL substrate (Tanon) was used for detection by MiniChmei Chemiluminescence imager (SAGECREATION, Beijing).

### Immunofluorescence

Cells were seed into confocal dish (NEST) and fixed with 4% paraformaldehyde (PFA) for 10 min at room temperature. After permeabilization with 0.5% Triton X-100 for 10 min, cells were blocked with 5% goat serum and incubated with primary antibody overnight at 4 °C. The next day, cells were washed and incubated with secondary antibody at room temperature for 1 h, and then were labeled the nuclei by using Hoechst 33342 for 5 min.

### Label-free quantitative mass spectrometry

Liquid chromatography with tandem mass spectrometry data from three technical replicates of exosomes derived from cells expressing Rab22a-NeoF1 or Vector were analyzed using MaxQuant (version 1.5.0.30), MS data were searched against the UniProtKB *Escherichia coli* database (2,585,998 total entries, downloaded 06/07/12). An initial search was set at a precursor mass window of 6 p.p.m. The search followed an enzymatic cleavage rule of Trypsin/P and allowed maximal two missed cleavage sites and a mass tolerance of 20 p.p.m. for fragment ions. Carbamidomethylation of cysteines was defined as a fixed modification, while protein N-terminal acetylation and methionine oxidation were defined as variable modifications for database searching. The cutoff of global false discovery rate for peptide and protein identification was set to 0.01. Protein abundance was calculated on the basis of the normalized spectral protein intensity (LFQ intensity).

### Electron microscopy

For verification of the purified exosomes using electron microscopy, the isolated exosomes suspended in PBS were dropped on formvar carbon-coated nickel grids. After staining with 2% uranyl acetate, grids were air-dried and visualized using a JEM-1400 electron microscope (JEOL Ltd., Japan) transmission electron microscope.

### Nanoparticle tracking analysis

The size and concentration of the purified exosomes were determined by using Particle Metrix (ZETA VIEW), which is equipped with fast video capture and particle-tracking software.

### Cell co-culture

ZOS-M-shNC or ZOS-M-sh*RAB22A*-NeoF1 were plated in 0.4 μm porous Transwell inserts (Corning Inc., Corning, NY, USA) suspended over 143B-Luc cells plated at the 1:1 ratio and co-cultured for 5 days. Then, 143B-Luc cells were collected and subjected to migration and invasion assays

### Migration and invasion assays

The 24-well Boyden chambers with 8-μm inserts coated with Matrigel (invasion) or without (migration). A total of 5 × 10^4^ cells per well were plated on the inserts and cultured at 37 °C in the upper chambers without serum. After 12 h, cell inserts were fixed with 4% PFA for 10 min, followed by PBS wash and hematoxylin staining to allow visualization and counting

### RhoA activation assay

For RhoA activation assay in vitro, the cells treated with exosomes (10 μg) for 1 h and were lysed according to the manufacturer’s protocol (cytoskeleton, RT02). The cell lysates were pulldown by glutathione *S*-transferase-Rhotekin-Rho-binding domain protein beads and were subjected to Western blotting.

### Cell-penetrating peptide iRGD block assay

The 293T cells were co-transfected with Rab22a-NeoF1-SFB and PYK2-V5 plasmids and then treated with 5 μg of 1–10 a.a.-iRGD peptide or random-iRGD for 24 h. The lysates were pulled down using anti-Flag beads or anti-V5 beads, and then were subjected to Western blotting. The same procedure was followed for pulldown Rab22a-NeoF1 using mono-antibody RAD5 in ZOS-M cells.

Stable cells cultured with 10% EV-depleted FBS were treated with 10 μM of 1–10 a.a.-iRGD peptide or random-iRGD for 24 h, exosomes were collected and then subjected to Western blotting, migration, and invasion assay.

### Animal studies

Animal studies were approved by the Animal Research Committee of Sun Yat-sen University Cancer Center. Male athymic BALB/C nude mice (4 weeks old) were obtained from the Vital River Laboratory Animal Technology (Beijing, China). The mixed cells (5 × 10^5^ 143B-Luc cells with 5 × 10^5^ ZOS-M-shNC or ZOS-M-sh*RAB22A*-NeoF1 cells, 1 × 10^6^ U2OS/MTX-Luc cells with 1 × 10^5^ ZOS-M-shNC or ZOS-M-sh*RAB22A*-NeoF1 cells, 1 × 10^6^ U2OS/MTX-Luc cells with 2 × 10^5^ ZOS-M-shNC or ZOS-M-sh*RAB22A*-NeoF1 cells) were co-injected orthotopically into the bones of mice, as previously described.^43^ After 6 weeks, lung metastases of 143B-Luc cells or U2OS/MTX-Luc cells in mice were determined.

Lung metastasis was determined by luciferase-based noninvasive bioluminescence imaging using the platform of IVIS Lumina II (PerkinElmer) and measured by hematoxylin and eosin staining on lung paraffin sections.

To analyze the role of CM or exosomes containing Rab22a-NeoF1 fusion protein in tumor metastasis, 4- to 6-week-old BALB/C nude mice were injected in 1 × 10^6^ 143B-Luc or U2OS/MTX300-Luc cells. Seven days later, CM (300 μL) or exosomes (10 μg) derived from the indicated stable cells were injected three times a week for 3 weeks. Metastases were evaluated by the IVIS Spectrum system (Caliper, Xenogen) and the lungs were collected for analysis.

For experimental CM and exosome education, CM (300 μL) and exosomes (10 µg) were intravenously injected every other day for 3 weeks, mimicking continuous and systemic exosome release. One day after the last treatment, mice were orthotopically injected with 1 × 10^6^ U2OS/MTX300-Luc cells or 143B-Luc cells in PBS.

For experimental clodronate liposome administration, mice co-transplanted U2OS/MTX300-Luc cells with ZOS-M cells at the 10:1 ratio (1 × 10^6^:1 × 10^5^) were intravenously injected with clodronate liposomes (100 μL) or an equal volume of PBS liposomes twice a week; after 3 weeks, lung metastases of U2OS/MTX-Luc cells in mice were determined. In exosome pre-educated model, mice were intravenously injected with clodronate liposomes (100 μL) or an equal volume of PBS liposomes twice a week combined with exosomes (10 μg) derived from 143B cells stably expressing the empty vector (Vector-exo) or Rab22a-NeoF1 (Rab22a-NeoF1-exo) every other day for 3 weeks. One day after the last treatment, mice were orthotopically injected with 1 × 10^6^ 143B-Luc cells in PBS.

### Peptide-blocking assay in vivo

The mixture of 1 × 10^6^ U2OS/MTX-Luc cells with 1 × 10^5^ ZOS-M tumor cells was co-injected orthotopically into the bones of mice. One week post injection, the 1–10 a.a.-iRGD and random-iRGD peptide (1 mg/kg) were injected via tail vein every other day. The lung metastasis of mice was evaluated by the IVIS Spectrum system (Caliper, Xenogen) and the lungs were collected for quantification analysis.

For the effects of these iRGD peptides on exosome-induced metastases, 4- to 6-week-old BALB/C nude mice were injected in 1 × 10^6^ 143B-Luc cells. Seven days later, exosomes (10 µg) derived from the indicated stable 143B cells under the treatment of either 1–10 a.a.-iRGD or random-iRGD were intravenously injected every other day for 3 weeks. Metastases were evaluated by the IVIS Spectrum system (Caliper, Xenogen) and the lungs were collected for analysis.

### Statistical analysis

All quantitative data are presented as the mean ± SD. The error bars indicate the SD. The GraphPad software was used for the statistical analysis. The unpaired *t* test was used to compare differences between two groups. One-way ANOVA tests were performed to compare differences between subgroups.

## Supplementary information

Supplementary Materials

## Data Availability

The data sets used for the current study are available from the corresponding author upon reasonable request.
