# Supplementary material for: Rab22a-NeoF1 fusion protein promotes osteosarcoma lung metastasis through its secretion into exosomes
Source: Signal Transduct Target Ther. 2021 Feb 11;6:59. doi: 10.1038/s41392-020-00414-1 (PMC7876000; doi:10.1038/s41392-020-00414-1)
Supplement: Supplementary file 1 — Supplementary Materials [file 41392_2020_414_MOESM1_ESM.pdf]

Supplementary Materials for

Rab22a-NeoF1 fusion protein promotes osteosarcoma lung metastasis through its secretion into exosomes

Li Zhong[1#], Dan Liao[1#], Jingjing Li[1#], Wenqiang Liu[2], Jingxuan Wang[2], Cuiling Zeng[1], Xin Wang[1], Zhiliang Cao[3], Ruhua Zhang[1], Miao Li[4], Kuntai Jiang[5], Yi-Xin Zeng[1], Jianhua Sui[3*], and Tiebang Kang[1*]

***Correspondence to:**

Dr. Tiebang Kang, kangtb@sysucc.org.cn.

Dr. Jianhua Sui, suijianhua@nibs.ac.cn

This PDF file includes:

Figures. S1 to S9

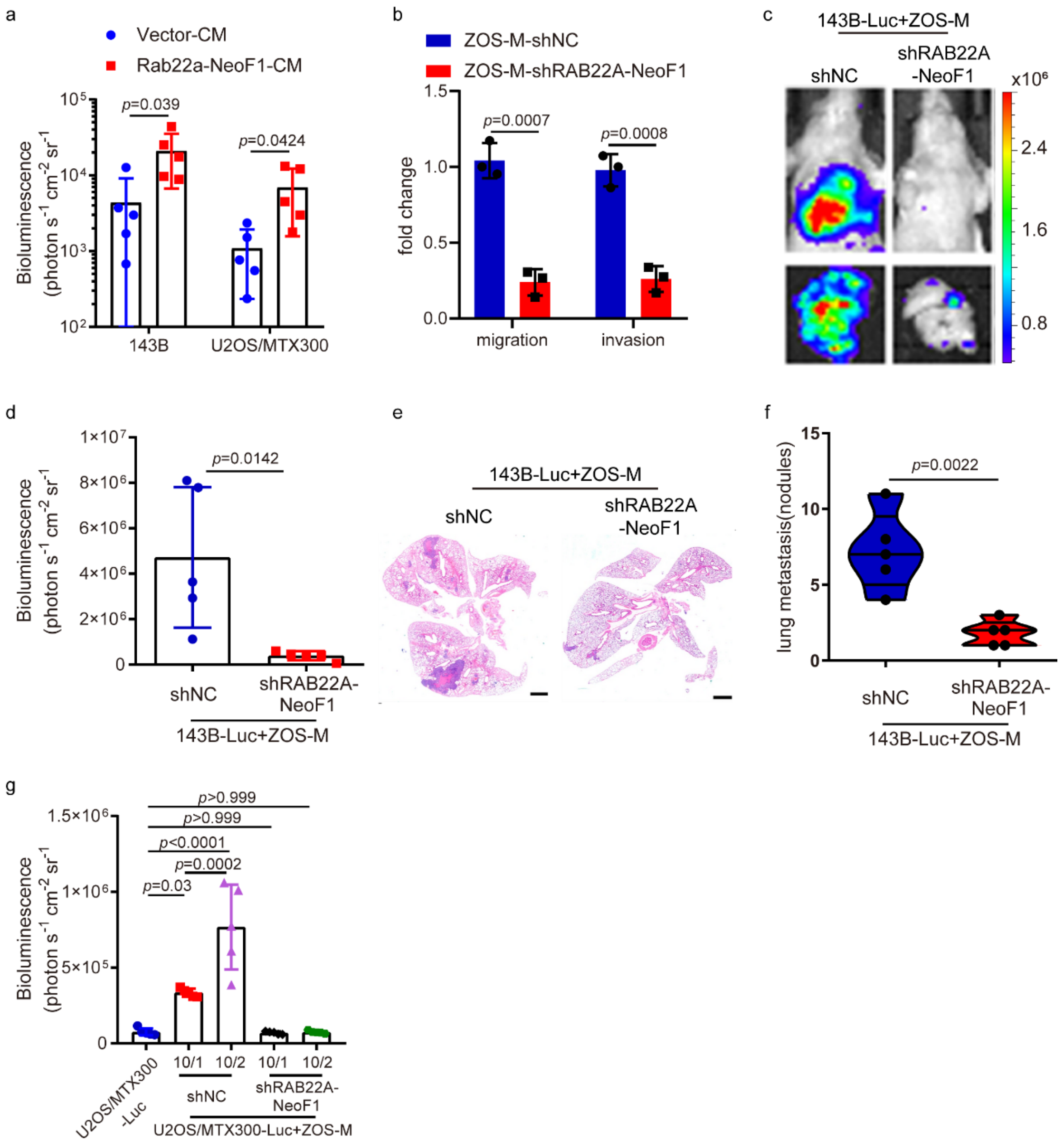

Figure. S1. The secreted RAB22A-NeoF1 fusion protein enhances metastasis in osteosarcoma.

(**a**) Bioluminescence analyses of lung metastases from mice orthotopically injected with the indicated cells under treatment of either Vector-CM or RAB22A-NeoF1 CM. n = 5 biologically independent mice. Data are mean ± s.d. *P* values are shown.

(**b**) 143B cells were co-cultured with the indicated stable cells for 5 days and then were subjected to migration and invasion assays. Data are mean ± s.d. of n = 3 biologically independent experiments. *P* values are shown.

(**c-f**) Representative IVIS imaging (**c**), Bioluminescence analyses (**d**)

H&E-stained lung sections (**e**) and quantification of lung metastatic foci (**f**) from mice orthotopically co-injected 143B-Luc cells with either ZOS-M-shNC cells or ZOS-M-shRAB22A-NeoF1 cells at the 1:1 ratio. n = 5 biologically independent mice. Data are mean ± s.d. *P* values are shown. Scale bar, 2mm.

(**g**) Bioluminescence analyses of lung metastases from mice orthotopically injected U2OS/MTX300-Luc cells alone or with either ZOS-M-shNC cells or ZOS-M-shRAB22A-NeoF1 cells at the indicated 10:1 and 10:2 ratios. n = 5 biologically independent mice. Data are mean ± s.d. *P* values are shown.

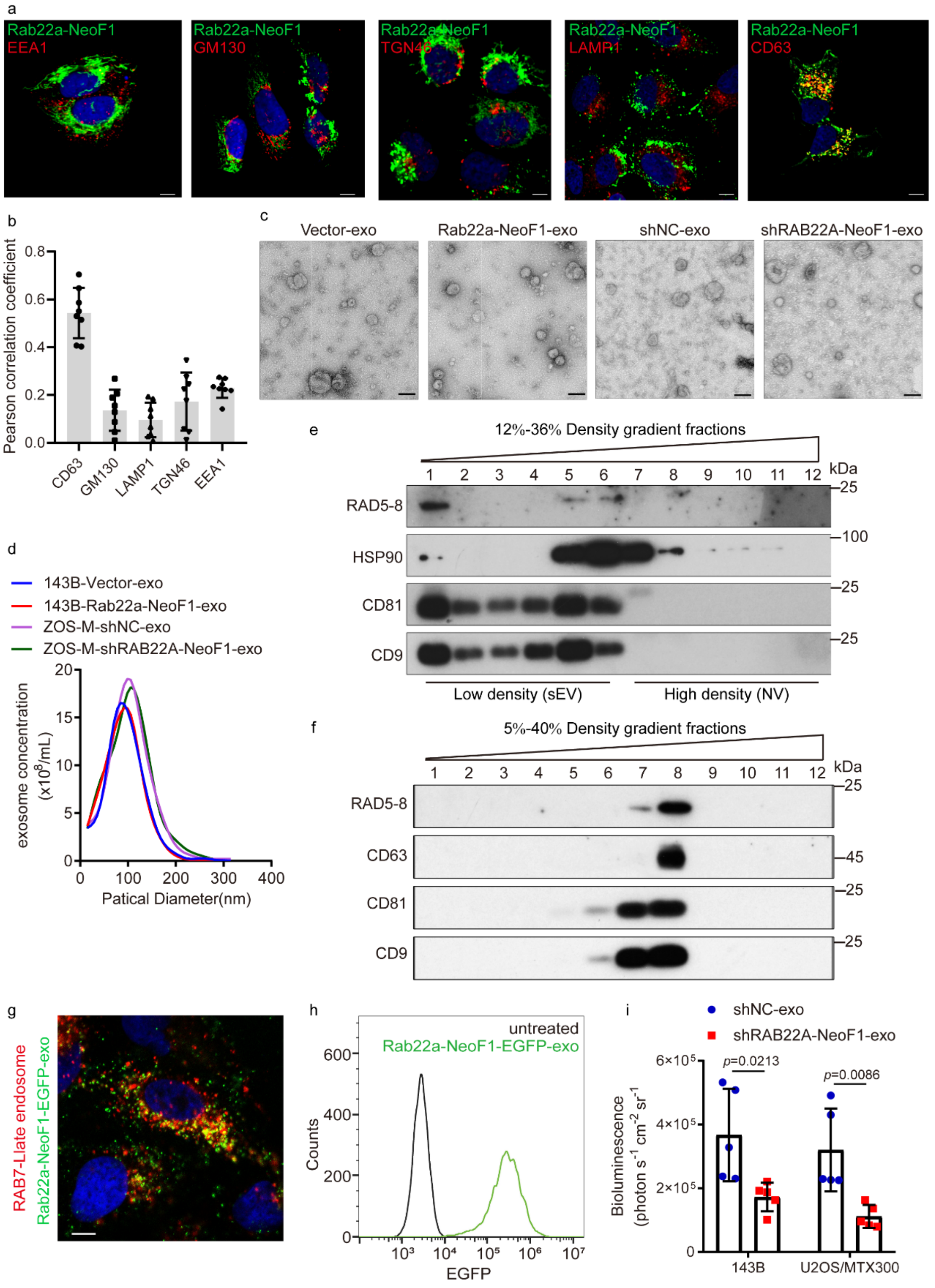

Figure. S2. The RAB22A-NeoF1 fusion protein is present in exosomes to promote osteosarcoma metastasis.

(**a**) Representative images of immunofluorescence staining for both intracellular RAB22A-NeoF1 and the indicated subcellular markers in U2OS cells stably expressing RAB22A-NeoF1. Scale bar, 5μm.

(**b**) Pearson correlation coefficients (PCCs) were calculated from multiple individual cells expressing RAB22A-NeoF1 and the indicated subcellular markers. mean ± SD. n = 8 fields. Data are mean ± s.d.

(**c**) Characterization of exosomes derived from the indicated stable 143B cells and stable ZOS-M cells using nanoparticle tracking analysis.

(**d**) Representative TEM images of the purified exosomes derived from the indicated stable 143B (up two panels) and stable ZOS-M cells (low two panels). Scale bar,100nm.

(**e, f**) 12-36% (**e**) and 5%-40% (**f**) iodixanol density gradients centrifugation was performed using the exosomes derived from 143B cells stably expressing RAB22A-NeoF1 as described in "Materials and Methods" and followed by Western blotting. Data in e and f are representative of n = 3 biologically independent experiments.

(**g**) Representative confocal microscopy image of U2OS cells treated with exosomes derived from 143B cells stably expressing Rab22a-NeoF1-EGFP (Rab22a-NeoF1-EGFP-exo) for 15min. The late endosomes (Red, denoted by RAB7) and GFP signals (Green) were shown. Scale bar, 5μm.

(h) The U2OS cells were treated with or without exosomes derived from 143B cells stably expressing Rab22a-NeoF1-GFP (Rab22a-NeoF1-EGFP-exo) for 15 min, and subjected to flow cytometry analysis.

(**i**) Bioluminescence analyses of lung metastases from mice orthotopically injected with the indicated cells under treatment of exosomes derived from the indicated stable ZOS-M cells. n = 5 biologically independent mice. Data are mean ± s.d. *P* values are shown.

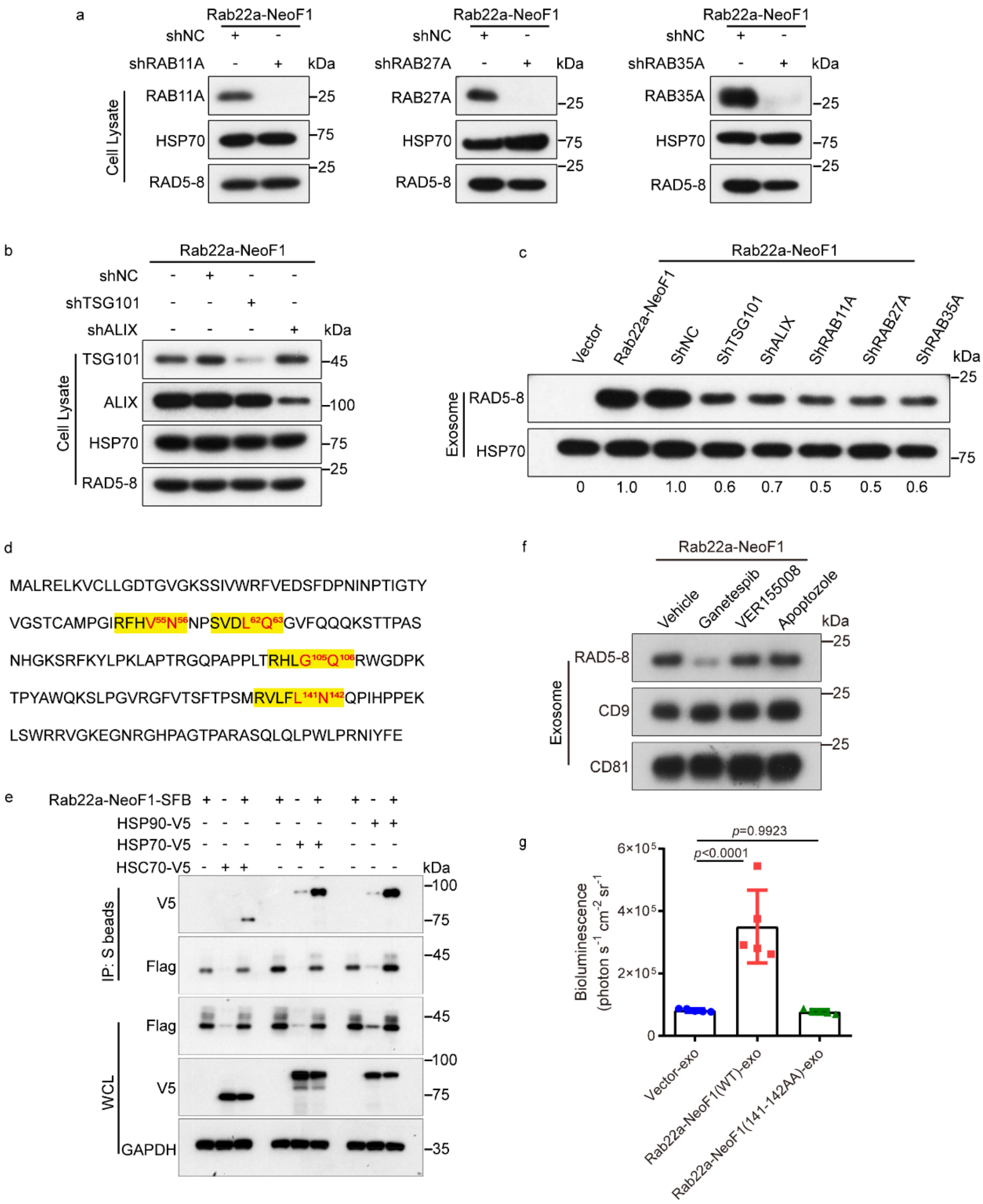

Figure. S3. RAB22A-NeoF1 fusion protein is sorted into exosomes via binding to HSP90 to promote osteosarcoma metastasis

(**a, b**) The indicated stable 143B cells were lysed and analyzed by Western blotting.

(**c**) The exosomes from the indicated stable 143B cells were purified and analyzed by Western blotting. The relative intensity of RAB22A-NeoF1 was quantified with ImageJ software and normalized with HSP70. Data in a-c are representative of n = 3 biologically independent experiments.

(**d**) The protein sequence of RAB22A-NeoF1. The yellow region indicates four KFERQ-like motifs (aa52-56, aa59-63, aa102-106 and aa137-142) and mutated amino acids are highlighted in red.

(**e**) 293T cells were co-transfected with the indicated plasmids and then were lysed and analyzed by immunoprecipitation using S protein beads and Western blotting.

(f) The 143B cells stably expressing Rab22a-NeoF1 were treated with the HSP90 inhibitor (Ganetespib), HSP70 and HSC70 inhibitors (VER155008 and Apoptozole) for 24h, and then the exosomes were purified and analyzed by Western blotting. Data in e and f are representative of $n = 3$ biologically independent experiments.

(**g**) Bioluminescence analysis of lung metastasis from mice orthotopically injected 143B-Luc cells with exosomes derived from the indicated stable 143B cells. $n = 5$ biologically independent mice. Data are mean ± s.d. *P* values are shown.

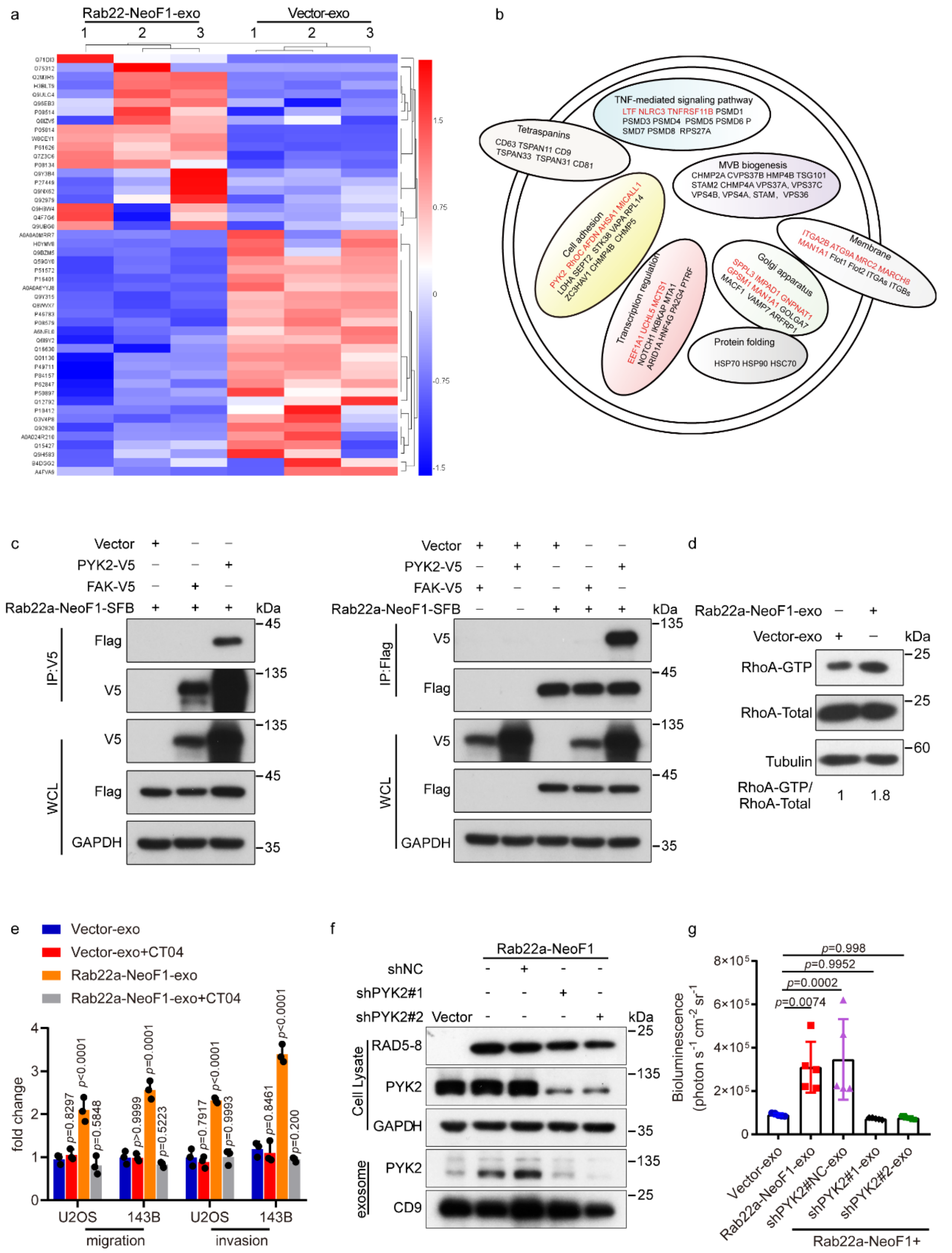

Figure. S4. The functions of exosomes containing RAB22A-NeoF1 fusion protein on its negative recipient cancer cells requires its binding partner PYK2 from donor cells.

(**a**) The heat maps of differential expression cluster in exosomes from the indicated stable 143B cells.

(**b**) The protein compositions in exosomes from the indicated stable 143B cells.

Proteins with significant increase from exosomes positive for RAB22A-NeoF1 were marked in red.

(**c**) 293T cells were co-transfected with the indicated plasmids and then were lysed and analyzed by immunoprecipitation using anti-Flag beads or anti-V5 beads and Western blotting.

(**d**) 143B cells treated with exosomes derived from the indicated stable 143B cells for 1 h, and then were subjected to the RhoA activation assay. The relative intensity of RhoA-GTP quantified with ImageJ software and normalized with RhoA-Total. Data in c and d are representative of n = 3 biologically independent experiments.

(**e**) U2OS and 143B cells were treated with or without RhoA inhibitor CT04 for 4h, and then were incubated with exosomes derived from the indicated stable 143B cells for 24h. The cells were then collected and subjected to migration and invasion assays. Data are mean ± s.d. of n = 3 biologically independent experiments. *P* values are shown.

(**f**) The exosomes and cell lysates were prepared from the indicated stable 143B cells, and then were analyzed by Western blotting. Data are representative of n = 3 biologically independent experiments.

(**g**) Bioluminescence analysis of lung metastatic foci from mice orthotopically injected 143B-Luc cells, and then were treated with the indicated exosomes. n = 5 biologically independent mice. Data are mean ± s.d. *P* values are shown.

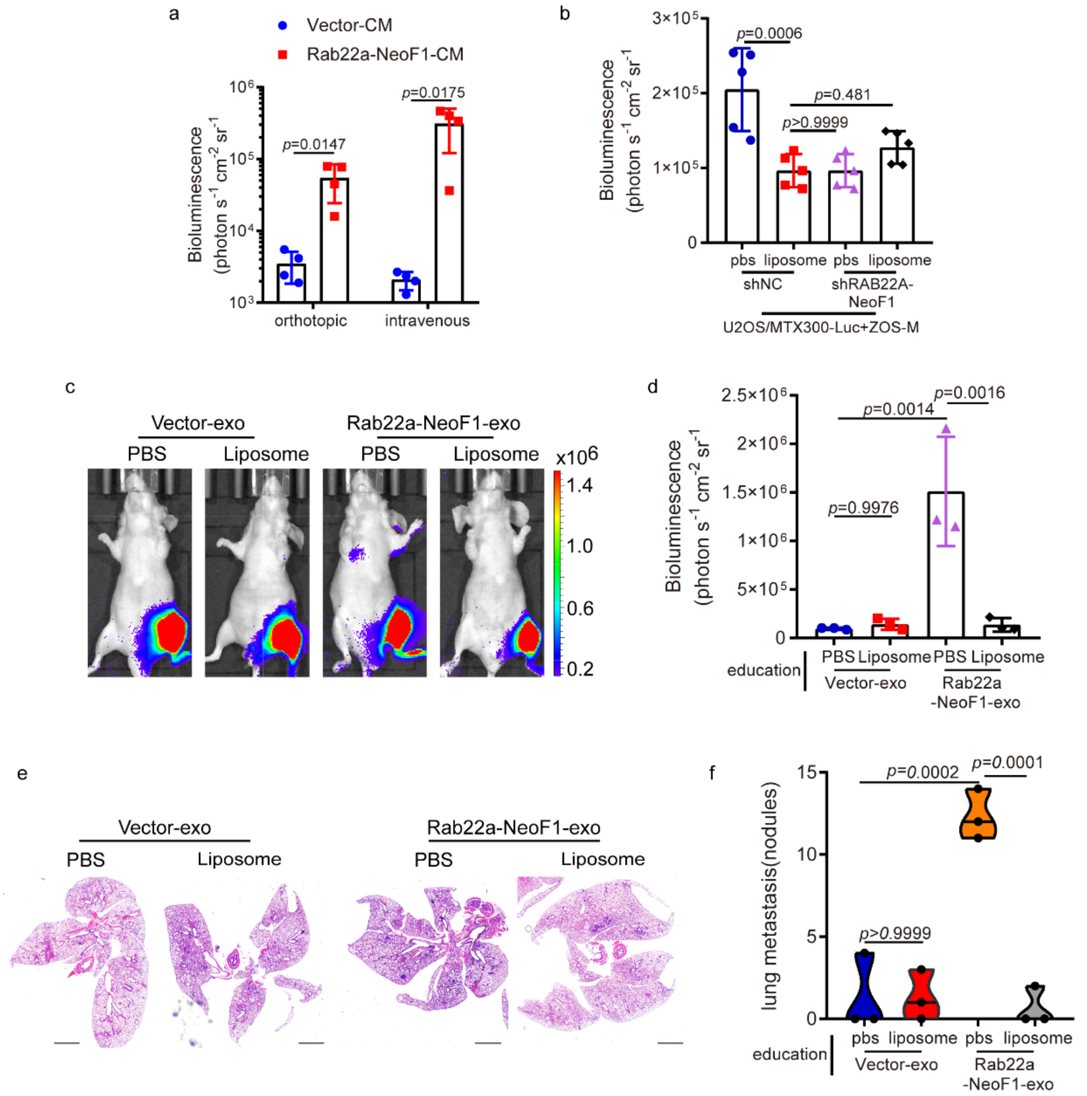

Figure. S5. The functions of exosomal RAB22A-NeoF1 fusion protein requires recruiting BMDMs to pulmonary pre-metastatic niche.

(**a**) Mice were pre-educated with the indicated conditioned media (CM) for 3 weeks, and then were orthotopically or tail vein injected with 143B-Luc cells. The lung metastases were analyzed by Bioluminescence after 3 weeks. n =4 biologically independent mice. Data are mean ± s.d. *P* values are shown.

(**b**) Mice orthotopically co-injected U2OS/MTX300-Luc cells with the indicated stable ZOS-M cells at the 10:1 ratio, and then intravenously injected liposome or PBS twice a week. The lung metastases were analyzed by bioluminescence. n = 5 biologically independent mice. Data are mean ± s.d. *P* values are shown.

(**c-f**) Mice were pre-educated with the indicated exosomes combined with liposome or PBS for 3 weeks, and then were orthotopically injected with

143B-Luc cells. The lung metastases were analyzed after 3 weeks. Representative IVIS imaging (**c**), Bioluminescence analysis (**d**), H&E-stained lung sections (**e**), Quantification of lung metastatic foci (**f**). n = 3 biologically independent mice. Data are mean ± s.d. *P* values are shown. Scale bar, 2mm.

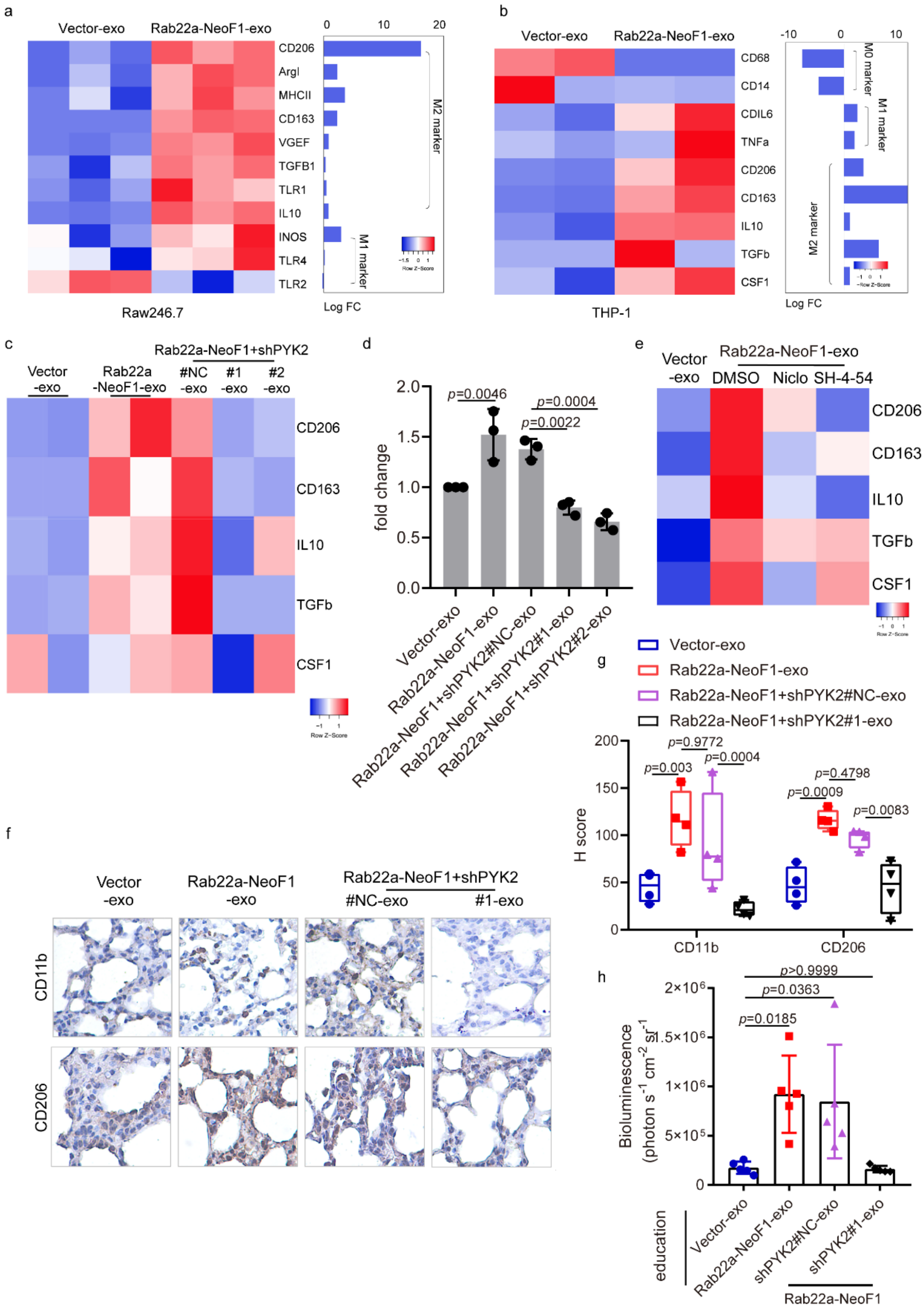

Figure. S6. The exosomal RAB22A-NeoF1 fusion protein promotes M2 polarization in its recipient macrophages via its binding partner PYK2. (**a, b**) Raw264.7 cells (**a**) and THP-1 cells (**b**) were incubated with the indicated exosomes for 24 h, and differential expression patterns of the M0, M1 and M2 phenotype markers were presented by a heatmap.

(**c**) THP-1 cells were incubated with the indicated exosomes for 24 h, and differential expression patterns of the M2 polarization macrophage markers were presented by a heatmap.

(**d**) Raw264.7 cells were incubated with the indicated exosomes for 24h, and were subjected to migration assays. Data are mean ± s.d. of n = 3 biologically independent experiments. *P* values are shown.

(**e**) Raw264.7 cells were treated the indicated exosomes with or without the indicated Stat3 inhibitor, Niclosamide or SH-4-54, for 24h and expression of M2-like macrophage markers were present by a heatmap.

(**f, g**) Mice lungs were pre-educated with the indicated exosomes for 3 weeks, and the CD11b and CD206 expression were analyzed by IHC. Representative staining imaging (**f**) and relative H-score of CD11b and CD206 (**g**). n = 4 biologically independent mice. Data are mean ± s.d. *P* values are shown.

(**h**) Mice were pre-educated with the indicated exosomes for 3 weeks, and then were orthotopically injected with 143B-Luc cells. The lung metastases were analyzed after 3 weeks. The lung metastases were analyzed by bioluminescence. n = 5 biologically independent mice. Data are mean ± s.d. *P* values are shown.

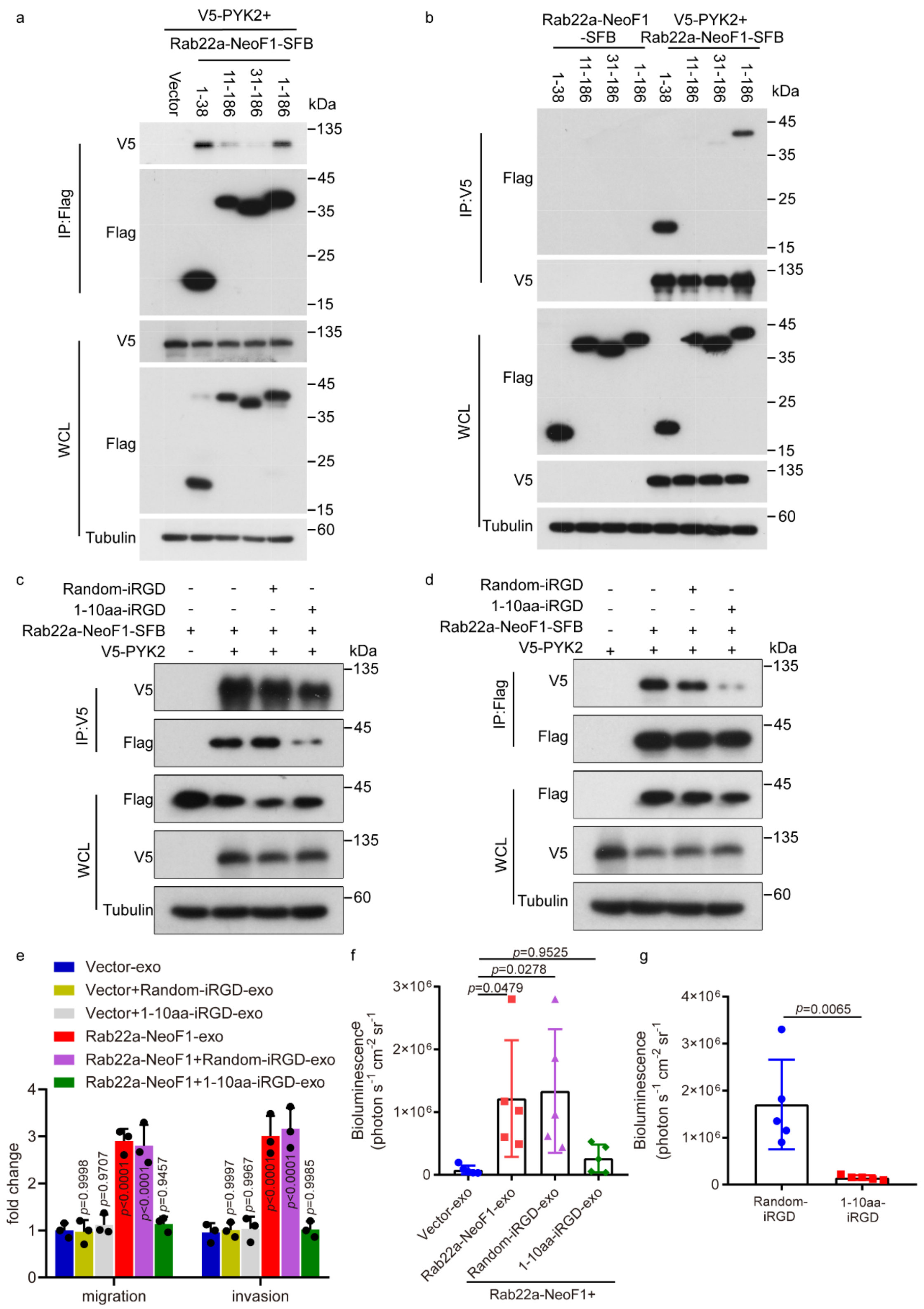

Figure. S7. The 1-10aa of RAB22A-NeoF1 is responsible for its binding to PYK2.

(**a, b**) 293T cells were co-transfected with the indicated plasmids and then were lysed and analyzed by immunoprecipitation using anti-Flag beads (**a**) or

anti-V5 beads (**b**) followed by Western blotting.

(**c, d**) 293T cells were co-transfected with the indicated plasmids and treated with the indicated peptide for 24 h, and then were lysed and analyzed by immunoprecipitation using anti-V5 beads (**c**) or anti-Flag beads (**d**) followed by Western blotting. Data in a-d are representative of n = 3 biologically independent experiments.

(e) U2OS cells were treated with the indicated exosomes for 24h, and then were subjected to migration and invasion assays. Data are mean ± s.d. of n = 3 biologically independent experiments. P values are shown.

(f) Bioluminescence analysis of lung metastatic foci from mice orthotopically injected 143B-Luc cells with the indicated exosomes. n = 5 biologically independent mice. Data are mean ± s.d. P values are shown.

(**g**) Bioluminescence analysis of lung metastatic foci from mice orthotopically co-injected U2OS/MTX300-Luc cells with ZOS-M cells at the 10:1 ratio under the treatment of either 1-10aa-iRGD or random-iRGD peptide. n = 5 biologically independent mice. Data are mean ± s.d. *P* values are shown.

a

Rab22a-NeoF2: MALRELKVCLLGDTGVGKSSIVWRFVEDSFDPNINPTIGTVHNIKGTNPDAYQ

Rab22a-NeoF3: MALRELKVCLLGDTGVGKSSIVWRFVEDSFDPNINPTIGFLILEGSCCPSYLRG

Rab22a-NeoF4: MALRELKVCLLGDTGVGKSSIVWRFVEDSFDPNINPTIGWSFTLVIPAGMQWHDLGSL
QPPPPGFKQFACLSLLRSWNYRCSQPHLANFCSFSRDGVSPCWPGWSRTPDLR

Rab22a-NeoF5: MALRELKVCLLGDTGVGKSSIVWRFVEDSFDPNINPTIGSRVQPCPWPFFGQLVVGDWFRYGHMT

Rab22a-NeoF6: MALRELKVCLLGDTGVGKSSIVWRFVEDSFDPNINPTIGFKPIFPKDQIGISI

b

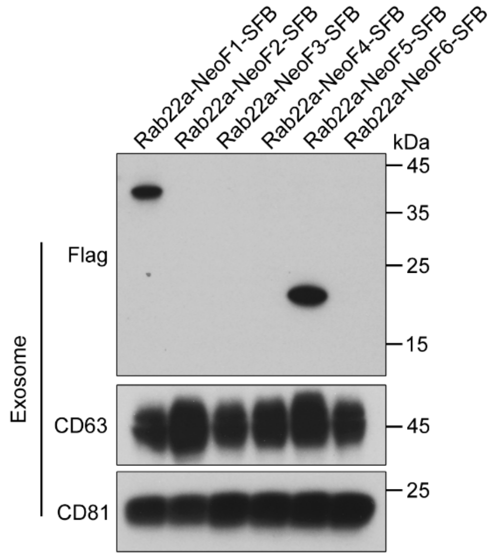

Figure. S8. Secretion of RAB22A-NeoFs proteins

(**a**) The protein sequences of RAB22A-NeoF2-6. The yellow region indicates the KFERQ-like motif (aa52-57).

(**b**) The exosomes derived from the indicated stable 143B cells were purified and subjected to Western blotting. Data are representative of n = 3 biologically independent experiments.

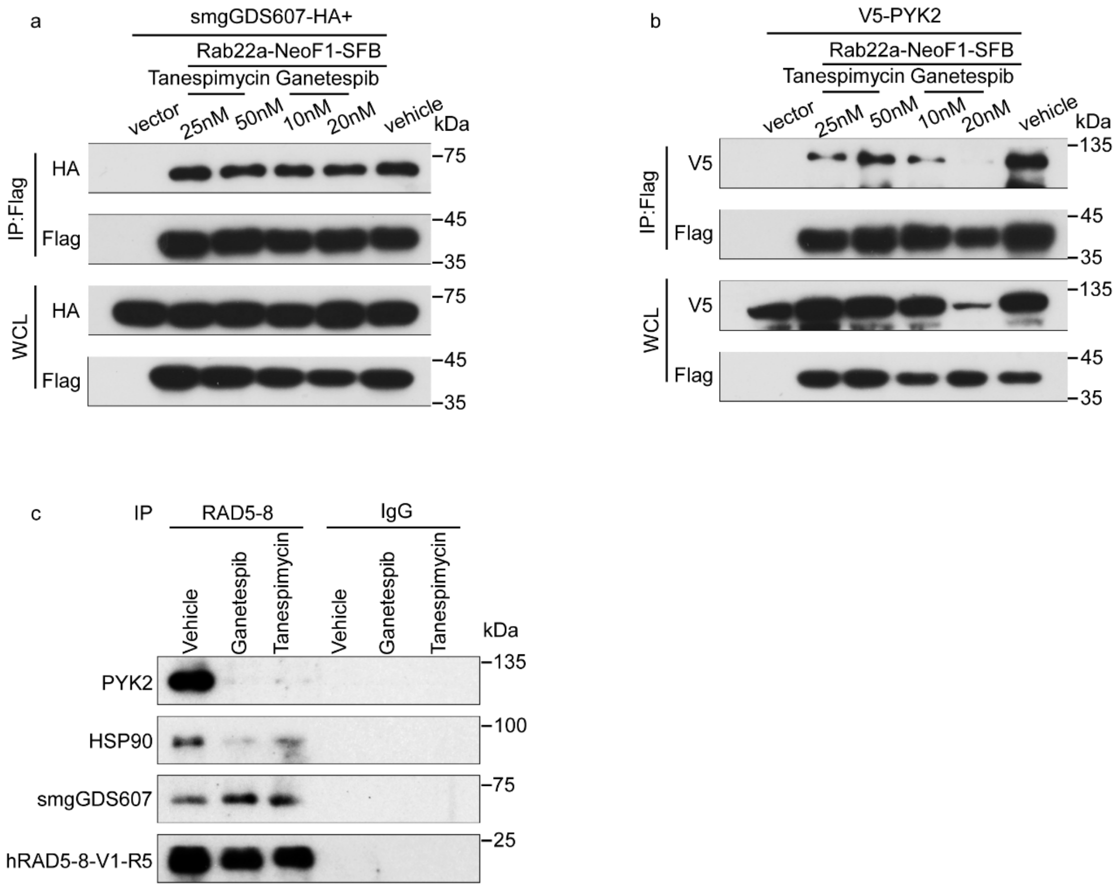

Figure. S9. The interaction of RAB22A-NeoF1 fusion protein with PYK2, but not with smgGDS607, is dependent on its binding to HSP90.

(**a, b**) 293T cells were co-transfected with the indicated plasmids and treated with the indicated HSP90 inhibitors for 24 h, and then were lysed and analyzed by immunoprecipitation using anti-Flag beads followed by Western blotting.

(**c**) ZOS-M cells were treated with the indicated HSP90 inhibitors for 24 h and then were lysed and subject to immunoprecipitation using IgG and mAb RAD5-8 followed by Western blotting. Data in a-c are representative of n = 3 biologically independent experiments.
